# A Smartphone Game to Prevent HIV Among Young Africans (Tumaini): Assessing Intervention and Study Acceptability Among Adolescents and Their Parents in a Randomized Controlled Trial

**DOI:** 10.2196/13049

**Published:** 2019-05-21

**Authors:** Gaëlle Sabben, Victor Mudhune, Ken Ondeng'e, Isdorah Odero, Richard Ndivo, Victor Akelo, Kate Winskell

**Affiliations:** 1 Hubert Department of Global Health Rollins School of Public Health Emory University Atlanta, GA United States; 2 Centre for Global Health Research, HIV Research Branch Kenya Medical Research Institute Kisumu Kenya

**Keywords:** HIV, youth, sub-Saharan Africa, Kenya, serious game, narrative, smartphone, pilot study, randomized controlled trial, acceptability

## Abstract

**Background:**

Young people aged 15 to 24 years account for one-third of new adult HIV infections. Controlling the HIV epidemic requires effective interventions targeted toward young people and their needs. Smartphone games offer a promising avenue for reaching this population with evidence-based HIV prevention interventions. It is crucial to the effectiveness of these interventions that they be acceptable and intrinsically motivating to adolescents as well as acceptable to their parents.

**Objective:**

*Tumaini* is a narrative-based smartphone game designed to help prevent HIV among young Africans aged 11 to 14 years by delaying first sex and increasing condom use at first sex. Following a 16-day feasibility study of *Tumaini*, we assessed the acceptability (1) of the intervention, where acceptability was operationalized as appeal, relevance, value, usability, and understandability, and (2) of this study and a planned future randomized controlled efficacy trial.

**Methods:**

During the randomized feasibility study (n=60) of *Tumaini* in western Kenya in spring 2017, 30 participants used the intervention on a study-provided smartphone. The app automatically logged participant interaction with the game in time-stamped log files. All 30 participants completed an Audio Computer-Assisted Self-Interview–based game experience survey, and 27 took part in 4 focus group discussions (FGDs) about the game’s appeal, relevance, value, usability, and understandability. Their parents (n=22) also participated in 4 FGDs about the acceptability of the intervention, of this study, and of a planned efficacy trial. Survey data were analyzed using SAS software (SAS Institute Inc); FGD transcripts were coded and analyzed in MAXQDA 12 (Verbi GmbH); and gameplay log files were analyzed using Microsoft Excel.

**Results:**

Adolescent participants’ survey responses indicated that *Tumaini* scored well with players on all indicators of acceptability (appeal, relevance, value, usability, and understandability). Focus group analyses aligned with these findings and emphasized a high degree of player engagement with the game, which was supported by log file analysis. Adolescent participants were eager for additional content, and parents were receptive to a longer study involving biomarkers, based on their positive experiences with this study. There is scope to improve communication with parents about their role in the intervention. As the game was tested in beta version, there is also scope to fine-tune some of the game mechanics to increase usability.

**Conclusions:**

This study shows the strong acceptability of an interactive smartphone-based game both to adolescents and their parents in western Kenya and that of the study methods used to pilot-test the intervention. It also suggests that longitudinal efficacy studies of this type of intervention, including those using biomarkers, have the potential to be acceptable among parents.

**Trial Registration:**

ClinicalTrials.gov NCT03054051; https://clinicaltrials.gov/ct2/show/NCT03054051 (Archived by WebCite at http://www.webcitation.org/70U2gCNtW)

## Introduction

### Background

Young people aged between 15 and 24 years, particularly young women and those from sub-Saharan Africa, account for a large proportion of new HIV infections among adults [1]. Due to demographic change, a youth bulge, or a particularly large cohort of young people aged 15 to 24 years, makes up a large segment of the population in many sub-Saharan African countries, including those affected by HIV [2]. Without a significant decrease in HIV incidence, this youth bulge will result in large numbers of new HIV infections among young people each year. Any effort to reduce the incidence and prevalence of HIV must address this age group’s specific needs through targeted prevention efforts. Reaching them with prerisk interventions can help them to delay sex and to develop safer sexual behaviors from the onset of sexual activity [3,4], including by establishing patterns of consistent condom use [5].

Mobile technologies, including smartphones, are rapidly becoming more affordable and accessible, including in sub-Saharan Africa [6,7]. Their increasing ubiquity offers exciting opportunities for delivering targeted, culturally relevant prevention messaging and skills-building interventions at scale and remotely [8]. This is especially appealing for settings where local capacity for implementation and facilitation of group-based interventions may be limited: remotely delivered interventions would not rely on trained facilitators to ensure intervention fidelity or to regularly provide updated content [9].

Smartphones provide a platform for interactive and immersive interventions, including games, which may be particularly well suited for young people [10] and have the potential to be leveraged in ways that cannot be achieved with traditional intervention models [11]. Serious games, that is, those designed with a primary purpose that is not entertainment [12], adequately grounded in behavioral, instructional, and communication theory [13-15], can provide an engaging and safe environment to build and rehearse health protective knowledge and skills. Studies of existing serious games have shown their effects on clinical outcomes among adolescents [14,16], and an emphasis is being placed on assessing the efficacy of newly developed technology-based interventions on both health and behavior [11].

Establishing intervention efficacy is crucial; however, it is no less important to assess factors likely to affect adoption, preferably early in the development process [9]. These factors include the acceptability of the intervention to its intended users, their intrinsic motivation to engage with it, and any potential barriers to its uptake [17]. As a key component of implementation research, appropriateness of an intervention can be defined as its “perceived fit, relevance, or compatibility” for a particular purpose and consumer [17]. For an intervention delivered using a technological platform, acceptability of the delivery mode itself, including its perceived ease of use and relevance, must be considered as influencing users’ eventual uptake [18]. In this paper, these aspects of user acceptance and perception of the intervention are combined under the umbrella term *acceptability*.

An intervention aimed at minors, especially one intended to be delivered remotely without a facilitator, must be acceptable to the young users as well as to their parents, who are likely to control access. Assessing intervention acceptability is particularly important when using new modes of delivery because little prior research exists to indicate what children and their parents are likely to find most palatable. In the case of interventions intended for low-resource settings, evaluation and dissemination of technologically advanced interventions and systems have received even less attention.

The success of an efficacy trial for an intervention aimed at minors also lies in the acceptability of the trial’s design to parents because their consent is necessary for children’s participation and thus the feasibility of the study. Parents can justifiably force study procedures to change or even a trial to end if the methods are unacceptable to them. Therefore, their acceptance of the methods is critical to studies’ success, in particular, that of studies focusing on adolescent sexual health, including HIV prevention research [19]. As such, parents must be involved early in the process of developing study methods to identify potential barriers to a study’s acceptability and smooth the way for successful efficacy trial implementation.

### Intervention

*Tumaini* is a narrative-based smartphone game designed to help prevent HIV among young Africans. It was tested in a randomized controlled feasibility trial in Kisumu, western Kenya, in collaboration with the Kenya Medical Research Institute (KEMRI) in spring 2017. Pilot outcome data from this study were promising, showing significant effects on behavioral mediators of sexual debut and condom use [20]. The objectives of the pilot study included assessing the acceptability (1) of the game-based intervention, where acceptability is defined as appeal, usability, understandability, relevance, and value and (2) of the study itself and of a potential future randomized controlled trial to establish the efficacy of the game to delay first sex and increase condom use at first sex. This planned study would test participants for HIV and herpes simplex virus type 2 (HSV-2) as proxy for cumulative sexual activity, a measure motivated by the recognized challenges with inconsistent self-reporting of sexual behavior (as a risk factor for HIV) among adolescents [21]. This paper thus shares findings on the acceptability to adolescent participants and their parents of the *Tumaini* intervention and the study itself with a view to informing the development of other related interventions and studies.

## Methods

### Data Collection

The participants allocated to the intervention arm of the study (n=30) were provided with low-cost Android smartphones loaded with the game *Tumaini* and asked to play for at least 1 hour per day for the duration of the 16-day study. All other phone functions, with the exception of the alarm clock (for reminders), were disabled. The app automatically collected time-stamped gameplay data. Phones were provided to participants for the duration of the study to ensure consistency of technology and to avoid any socioeconomic bias resulting from using smartphone ownership as a criterion for inclusion in the study.

Both adolescents and their parents were told that *Tumaini* was the adolescents’ “space to learn” and that adolescents should feel free to navigate the game as they wished. It was explained that adolescents could seek help or advice from and speak to others about the game and its contents if they so desired, and that parents should feel comfortable speaking to their children about the game and what he or she was learning. Parents were also asked to remind their children to play daily and to contact the study staff with any concerns, problems, or questions.

Immediately postintervention, all 30 participants completed a survey about their experience playing the game, delivered via Audio Computer-Assisted Self-Interview. Participants and their parents were also invited to take part in focus group discussions (FGDs) to provide additional insight into their experiences during the study. Ethical approval was granted by the Emory University and KEMRI institutional review boards, and the study was registered with ClinicalTrials.gov. Further details of the study protocol are provided elsewhere [22].

### Intervention

*Tumaini* aims to help prevent HIV among young Africans by delaying first sex and increasing condom use at first sex. It does this by increasing knowledge about sexual health and HIV; building risk-avoidance and risk-reduction skills and related self-efficacy; challenging HIV stigma and harmful gender norms and attitudes; fostering future orientation, goal setting, and planning; and promoting dialogue with adult mentors. *Tumaini* was developed in collaboration with a US commercial game developer, Realtime Associates, and US-based and Kenyan specialists in adolescent sexual health and HIV prevention and with input from Kenyan adolescents and their parents. The game is in English with an audio track featuring Kenyan voice talent.

*Tumaini* is grounded in narrative and narrative-based applied communication and social behavioral theory and existing evidence-based HIV prevention interventions to promote problem solving, cognitive and behavioral rehearsal, observational learning, and immersion (manuscript under review), and it draws on extensive research on HIV-themed narratives written by young Africans [23-25].

The choose-your-own-adventure interactive narrative follows 6 characters, 3 male and 3 female, as they progress through adolescence and face real-life challenges that players are likely to face in their own lives. These challenges include peer pressure, puberty, violence, and decisions about smoking, alcohol, drugs, and sex. Players make decisions for the characters that affect their narrative trajectories and reflect possible real-world consequences of these decisions. The content explored in the narrative is bolstered by 2 additional components: minigames that tie into the themes of the narrative chapters and strengthen players’ knowledge and skills and *My Story* in which players create a personal avatar and respond to questions that connect the contents of the role-playing narrative to their lives. *Tumaini* includes approximately 12 hours of discrete gameplay; it is designed to be replayed so that players can observe the outcomes of different decisions.

### Participants

All 30 intervention arm participants played the game and responded to the survey. Subsequently, 27 participated in 4 FGDs, stratified by participant gender and age (11-12 and 13-14 years). The 4 parent FGDs were stratified by gender and age of the enrolled child and included 22 parents, mostly female. The demographics of the players, adolescent FGD participants, and parents are presented in Table 1.

**Table 1:** Participant demographics.

Characteristics		Intervention adolescents (n=30)	FGD^a^ adolescents (n=27)	FGD parents (n=22)
**Gender, n (%)**				
	Female	14 (47)	12 (44)	19 (83)
	Male	16 (53)	15 (56)	3 (17)
Age (years), mean (SD)		12.8 (1.0)	12.9 (0.9)	—^b^
**Religion, n (%)**				
	Catholic	14 (47)	13 (48)	—
	Protestant/Anglican	8 (27)	6 (22)	—
	Muslim	2 (7)	2 (7)	—
	Seventh-day Adventist	4 (13)	4 (15)	—
	Other	2 (7)	2 (7)	—
Living with both parents, n (%)		22 (73)	19 (70)	—
**Housing type, n (%)**				
	Permanent	8 (27)	8 (30)	—
	Semipermanent	11 (37)	9 (33)	—
	Temporary	9 (30)	8 (30)	—
	Iron sheets	2 (7)	2 (7)	—
**Smartphone ownership (check all that apply), n (%)**				
	Parent	21 (70)	19 (70)	—
	Self	2 (7)	2 (7)	—
	Sibling	11 (37)	10 (37)	—
	Other adult	4 (13)	4 (15)	—
	No one	3 (10)	3 (11)	—
Have used a smartphone before baseline, n (%)		22 (73)	20 (74)	—

^a^FGD: focus group discussion.

^b^Demographic data not collected directly from parents but expected to reflect adolescents’ self-report.

### Measures and Analysis

The Technology Acceptance Model’s (TAM) framework for the acceptability of technology-based systems separates acceptability or *user acceptance* into (1) a system’s usefulness and (2) its ease of use. These influence a user’s *affective response* and, as a result, likelihood of using the system [18]. In line with this framework, the survey included questions on the game’s appeal, relevance and value (usefulness), and usability and understandability (ease of use). The appeal of specific aspects of the game, including its graphics and characters, was also assessed, and as an indirect measure of likability, participants were asked whether they would recommend the game to a friend. Ease-of-use measures focused on how easy it was to understand how to play the game (usability) and to understand the English used in the game (understandability). The game’s usefulness questions focused on what players had learned, how useful they found that content (value), and how suitable they found the game to be for different player age groups and genders (relevance).

The FGD guide for adolescents included questions about players’ impressions of the game, parts they liked and did not like, and sections that were hard to play; individual game components; what they learned; and recommendations for additional changes or other comments. The discussion guide for parents focused on their reactions to the game and study, their suggestions for improvements, and their recommendations for a future larger longitudinal study likely to involve blood tests. The focus groups were conducted in a mixture of English, Dholuo, and Kiswahili by moderators fluent in all 3 languages. The participants were encouraged to use any combination of languages they were most comfortable using.

Survey data were imported into and analyzed using SAS software (SAS Institute Inc), version 9.4. Descriptive statistics were calculated and stratified by participant sex and age group. Transcripts were translated into English and uploaded to MAXQDA 12 software (Verbi GmbH) for thematic coding using inductive (eg, *age appropriateness*) and deductive (eg, *misunderstandings*) codes. The data were analyzed thematically and compared across demographics. Once the phones were collected at the end of the intervention, the *Tumaini* app gameplay log files were exported to Microsoft Excel and analyzed for mean exposure time.

## Results

### Acceptability of the Game

#### Appeal

Survey responses indicated that most players played every day as instructed (87%, 26/30), in most cases for at least an hour each time (77%, 23/30; Table 2). Almost all players reported that playing was "very fun" (90%, 27/30), with the 3 others, all older male participants, responding that playing was “fun.” Most also responded that given the opportunity, they would like to play the game much more (93%, 28/30) and that they would tell friends to play (97%, 29/30), a measure of participants’ engagement with the intervention. Log file analyses indicate that players used the game for close to 27 hours on average, and all but 1 participant completed the game at least once.

Participants’ enthusiasm was apparent during both participant and parental focus groups. The adolescents explicitly and repeatedly described the game as "fun", with 1 young girl saying she “felt like laughing” when she played (FGD for 11- to 12-year-old females) and all groups saying they would tell their friends to play also. One older male participant noted that playing a variety of characters was very enjoyable:

I felt good playing them because even when I was watching TV and I found it boring, I would just go to my bed and look at the game

FGD for 13- to 14-year-old males

Participants identified the skills they learned and the future goal planning practice as especially motivating and useful. They suggested that the emphases on refusal skills, condom use, HIV prevention, and future planning would also be appealing to their friends. Parents’ comments about their children’s engagement with the game provided further indication that it had high motivational appeal. Children appear to have played “willingly,” not needing or relying on the alarm to remind them to play, and there were several spontaneous reports of children being reluctant to return the phones at the end of the study as they wanted to play more.

Also speaking to the game’s appeal to players, participants enthusiastically suggested adding further content to the game and expanding prizes in the game’s reward system. The older male participants were particularly enthusiastic about this, asking for “30 chapters,” “or even 55,” as well as more characters of both genders, growing up over a longer period.

When asked which aspect of the game they liked most (story, minigames, *My Story*, rewards, or look of the game), 14 of the 29 players who indicated a preference (48%) chose the story, although 29 (97%) players also reported liking the design and minigame components “a lot.”

#### Usefulness

Players were also enthusiastic about *Tumaini* ’s value to their lives and its relevance for their age group. All players found *Tumaini* to be equally suitable for male and female players (Table 3). When asked what age group the game was "best for", half of the players (n=15/30) selected the 13 to 14 years age range, and 12 others (40%) identified the 11 to 12 years age range. Two-thirds of the participants indicated that the game was best suited to their own age group (11-12 or 13-14 years).

##### Relevance

A total of 13 participants (43%, 13/30) further indicated that *Tumaini* would be suitable for adolescents over 14 years and 6 participants (20%, 6/30) for children under 11 years. However, during FGDs, 5 younger children expressed discomfort around the age-appropriateness of certain content: 2 boys and 1 girl said that because of their age they were "confused" about or "uncomfortable" with the reproductive system and information about condom use, and 2 girls reported being "frightened" by scenarios of older men pressuring young women (FGDs for 11- to 12-year-old males and females). Older participants, in contrast, appreciated playing older characters in preparation for encountering those situations in their own lives. As one 13- to 14-year-old male explained:

...it felt good because as I was playing the game I was imagining these things happening in real life.

FGD for 13- to 14-year-old males

Concerns about content age-appropriateness were expressed in one of the focus groups by 2 parents of younger children (FGD2 for parents of 11- to 12-year-old children). The mother of an 11-year-old boy felt that he should not yet be exposed to information about condoms. When asked if the subject should be removed, she demurred:

...it’s hard for a parent to teach them because it’s of no use to them […] the 11-12 year olds are still young and don’t even know how to use condoms

**Table 2:** Frequency and percentage of respondents responding most positively to questions on appeal by gender.

Variables	Age 11-12 years, n (%)		Age 13-14 years, n (%)		All (n=30), n (%)
	Males (n=7)	Females (n=5)	Males (n=9)	Females (n=9)	
Played every day	7 (100)	4 (80)	7 (78)	8 (89)	26 (87)
Played 1 hour or more each time	7 (100)	2 (40)	7 (78)	7 (78)	23 (77)
In general playing was very fun	7 (100)	5 (100)	6 (67)	9 (100)	27 (90)
Would like to play much more	6 (86)	4 (80)	9 (100)	9 (100)	28 (93)
Would tell friends to play	6 (86)	5 (100)	9 (100)	9 (100)	29 (97)

**Table 3:** Frequency and percentage of participant responses to questions on relevance of game by gender.

Variables		Age 11-12 years, n (%)		Age 13-14 years, n (%)		All (n=30), n (%)
		Males (n=7)	Females (n=5)	Males (n=9)	Females (n=9)	
**Age groups suitable for? (years; check all that apply)**						
	Younger than 9	0 (0)	2 (40)	1 (11)	0 (0)	3 (10)
	9-10	0 (0)	1 (20)	1 (11)	2 (22)	4 (13)
	11-12	4 (57)	5 (100)	7 (78)	3 (33)	19 (63)
	13-14	4 (57)	5 (100)	8 (89)	7 (78)	24 (80)
	15-16	1 (14)	2 (40)	5 (56)	3 (33)	11 (37)
	Older than 16	0 (0)	0 (0)	3 (33)	3 (33)	6 (20)
**Age group best for? (years)**						
	Younger than 9	0 (0)	1 (20)	0 (0)	0 (0)	1 (3)
	9-10	0 (0)	0 (0)	1 (11)	0 (0)	1 (3)
	11-12	5 (71)	3 (60)	2 (22)	2 (22)	12 (40)
	13-14	2 (29)	1 (20)	6 (67)	6 (67)	15 (50)
	15-16	0 (0)	0 (0)	0 (0)	1 (11)	1 (3)
	Older than 16	0 (0)	0 (0)	0 (0)	0 (0)	0 (0)
Best for both genders		7 (100)	5 (100)	9 (100)	9 (100)	30 (100)

Most parents, however, indicated that although they had some initial fears about the appropriateness of the content, these were soon allayed:

I was initially worried that there would be harm, that the game would contain sexual issues or even pornography that would influence her attitude...after playing the game for like a week, I stopped being concerned

FGD2 for parents of 13-14 year olds

One parent (FGD2 for parents of 13-14 year olds) soon realized that the game, rather than being a bad influence, actually “discouraged her from such bad thoughts about sex.”

##### Value

The game also scored high on the measures of its value to participants. All 30 participants indicated that they had learned “a lot” by playing *Tumaini* and that the information they had acquired would be “very useful for the future” (Table 4). All but 1 participant (97%, 29/30), a young male, also indicated that they had found the game’s contents to be immediately useful. Participants also felt that the game had increased their preparedness and self-efficacy for managing risky situations: 28 (93%, 28/30) participants responded that they felt more prepared to handle difficult situations, and 29 (97%, 29/30) responded that they were more sure that they could say no firmly in situations of pressure.

During FGDs, adolescents explained that an important aspect of the game’s intrinsic appeal lay in the value of the game and its content to their lives both in the immediate and in preparation for situations they might face in the future. Some reported feeling encouraged by characters’ positive developments and, in some cases, applying what they had learned in their own lives or sharing their new knowledge to help others. When discussing why they would recommend the game to others, 1 young boy summarized:

I would like to tell them that the game of Tumaini is a very beautiful game that you can play and understand things that may help you in life.

FGD for 11- to 12-year-old males

**Table 4:** Frequency and percentage of participants responding most positively to questions on value of game by gender.

Variables	Age 11-12 years, n (%)		Age 13-14 years, n (%)		All (n=30), n (%)
	Males (n=7)	Females (n=5)	Males (n=9)	Females (n=9)	
Learned a lot	7 (100)	5 (100)	9 (100)	9 (100)	30 (100)
Information very useful now	6 (86)	5 (100)	9 (100)	9 (100)	29 (97)
Information very useful for future	7 (100)	5 (100)	9 (100)	9 (100)	30 (100)
Since playing Tumaini, I feel more prepared for difficult situations I might face in the future	7 (100)	3 (60)	9 (100)	9 (100)	28 (93)
Since playing Tumaini, I feel more sure I can say no firmly when people are trying to pressure me	7 (100)	5 (100)	9 (100)	8 (89)	29 (97)

Parents also expressed satisfaction with the ways in which the content of the game related to the real-life challenges young people face. They reported that their children had shown increased interest in topics such as HIV after playing the game and that the lessons their children had shared with them were beneficial. These benefits ranged from recognizing and distancing themselves from peers’ negative influences to increasing empathy for others and effectively planning for the future. Part of the appeal of the game for parents was its resonance with their children’s lives:

...the game touched many things; school life, life at home, diseases like HIV...to me it was all okay because it touches all the parts of life.

FGD1 for parents of 13-14 year olds)

#### Ease of Use

The game’s usability and understandability scored reasonably well on the game experience survey, with 67% (20/30) of adolescents rating *Tumaini*"very easy to understand how to play" and a further 10% (3/30) rating it “a little easy” to understand (Table 5). Usability rated higher among older participants.

##### Usability

During FGDs, adolescents also gave a positive assessment of the game’s usability, with participants in all groups reporting that overall the game was "easy", both to play and understand. Most parents confirmed that their children had not needed much help to navigate *Tumaini*. Minor usability concerns centered on certain of the minigame mechanics being too complex, particularly among younger players, though players were able to overcome those hurdles by seeking advice from parents, siblings, or other trusted adults. Similarly, although adolescent participants in every FGD noted challenges that arose from content that they had not encountered previously, they succeeded by seeking help or, as a 13- to 14-year-old girl noted, through perseverance:

...some parts like drawing the condom steps were difficult but I tried and managed.

FGD for 13- to 14-year-old females

Parents in 2 FGDs mentioned having contacted the study staff for troubleshooting help. Although a few participants mentioned minor bugs that remained in the version of the game used for the trial, these do not seem to have affected players’ engagement with the game or its usability.

##### Understandability

The game’s level of English did not prove a challenge to players, with 83% (25/30) saying the English was “easy to understand” and 1 parent (FGD2 for parents of 11-12 year-olds) reporting that her child had told her that “the English in the game was simple, they are able to understand.” One parent (FGD2 for parents of 11-12 year olds) even reported that her son had asked the rest of the household to speak English as a result of playing the game. Indeed, in the survey, 25 participants (83%, 25/30) also said that English was the language in which they would most like to play, rather than Dholuo or Kiswahili. Of the others, 2 participants expressed a preference for Dholuo and 3 for Kiswahili.

The intervention showed a high level of acceptability to adolescents, judging by their positive reviews in terms of its appeal, value, and relevance. Participants found the game a highly appealing way to learn important information, would recommend the intervention to others, and, given the chance, opt to play longer. Adolescents also confirmed that, overall, *Tumaini* had been appropriately pitched to their age group, although they indicated that older adolescents might also enjoy and benefit from playing. The content of the game also spoke to the adolescents, who reported gaining knowledge, skills, and self-efficacy with both immediate and future relevance.

### Acceptability of the Study

#### This Study

Parents’ experiences with the pilot study appear to have been very positive. They thanked the team for engaging them in the study, and several spontaneously asked to be included in future studies. Most comments about the acceptability of the study related to the acceptability of the intervention and its positive influence on their children, either through its content or by keeping the child busy during the school holidays. Delivering the intervention via smartphone was also acceptable to parents, who noted the family and adolescents’ joy at having a phone to play with as part of the study. They appreciated that the phones’ other functions were blocked so that it could not be used for communication by their children. They had no concerns about their children’s safety while in possession of the phone, in part because the functions were blocked.

A small number of parents reported not having felt free to interact with their children around *Tumaini* and its content. Although many parents engaged with their children on a range of topics and with positive results, 3 parents noted that the study team’s instructions had discouraged them from playing the game or interacting with their children as they would have liked, with 1 parental FGD agreeing on this point when it was raised. These parents had understood the study team’s instructions about allowing the child to play without interference as restricting parents from responding to their children’s questions or helping their children navigate difficult parts of the game.

**Table 5:** Frequency and percentage of respondents responding most positively to questions on usability and understandability by gender.

Variables	Age 11-12 years, n (%)		Age 13-14 years, n (%)		All (n=30), n (%)
	Males (n=7)	Females (n=5)	Males (n=9)	Females (n=9)	
Very easy to understand how to play	4 (57)	3 (60)	7 (78)	6 (67)	20 (67)
English was easy to understand	4 (57)	4 (80)	8 (89)	9 (100)	25 (83)
Want to play game in English	6 (86)	4 (80)	7 (78)	8 (89)	25 (83)

In one focus group, 2 parents agreed that:

...there were some parts that when he reached he could have wanted to ask me, but from the way they were told to own the phone he could not ask

and that the study team:

...didn’t set us free to discuss with this kids when they were playing this game […] maybe if you left us free that maybe we can interact with them at the time they are playing that game it would have been fair.

FGD1 for parents of 11-12 year olds

Despite this misunderstanding, there were many reports of parent-child communication across a broad range of topics, which are detailed in a separate publication [26].

#### Future Studies

Parents discussed the possibility of future studies involving *Tumaini*, focusing on the logistics of a longer study, as well as the possibility of such a study including blood draws and HSV-2 testing. Parents cited their positive experience with this trial in support of a longer study and their expectation that any gains their children made during a short intervention period would be magnified if they were exposed to the game for longer. Several, in fact, volunteered their family’s participation in such a study.

In general, there was no opposition to blood collection or to HSV-2 testing. Several parents of younger children wanted to be present for blood draws and emphasized that communication from the study team about the purpose of the blood draw would be important to ensure acceptability both among potential participants and in their communities. One mother explained:

It’s important you educate so that we be open and free. If you don’t educate us it can be a flop because people have been doing monkey business with people’s blood, which has made people develop some diseases that they cannot explain.

FGD1 for parents of 11-12 year olds

Some also highlighted the importance of preparing the child for the test and for the possibility of positive results, including appropriate explanations, support, and counseling. They also expressed a desire to know the results of any test that was conducted.

Parents questioned when the study intervention periods and study activities would take place, whether during school or holiday periods. Most voiced a preference for the study to only take place during holidays so that it would not coincide with school responsibilities, and children would not be distracted from schoolwork by the game.

## Discussion

### Principal Findings

Both participants and their parents expressed a high level of acceptability for the game *Tumaini*. Adolescents and parents indicated that *Tumaini* held a great deal of appeal, was highly valuable and relevant to adolescents’ lives, and was overall easy to use and understand. Parents responded positively to a planned multiyear study involving biological biomarkers. These findings, in combination with the data on the intervention’s effects on secondary (behavioral) outcomes, presented in a separate publication [20], suggest that a large-scale efficacy trial of *Tumaini* is warranted and would be acceptable to parents of the target adolescent population.

#### Acceptability of the Intervention

Adolescents’ positive assessments of the interventions suggest that these types of serious games and smartphone-based intervention are acceptable and engaging ways to reach young people with important sexual health content, including about HIV. Whether the participants were motivated to use the intervention because of its entertaining nature or its educational promise, it is clear that acceptability was high. In the context of a self-regulated and remotely delivered intervention, such as this one, it is crucial that users be motivated to access and use the intervention repeatedly. This group of adolescent participants reported regular extended use of the game, requested additional time with the intervention, and suggested that additional content be made available, reflecting high intrinsic motivation to use the app. Parents’ reports that their children had played willingly and had not needed the alarm reminder to do so imply that *Tumaini’s* intrinsic motivational appeal to adolescents may be high enough to result in unprompted use by the target audience in the context of a longer efficacy trial and beyond.

Although acceptability is understood to be a key aspect of early intervention development [9], definitions of the term *acceptability* with regard to an intervention are inconsistent; reliable measures to assess this construct are few [27]. *Tumaini’s* nature as a technology-based intervention resulted in this study’s definition of acceptability drawing on Davis’s TAM framework [18], in addition to Proctor’s definitions of acceptability [17], to account for the game’s perceived usefulness to and ease of use for participants, both of which affect its overall likeability. Participants linking the appeal of *Tumaini* to its perceived usefulness and ease of use suggests that the TAM is a useful framework for assessing acceptability of this type of intervention. At the time of study design and measures development, there were, to the best of our knowledge, no validated acceptability scale or measures. However, the acceptability and appropriateness dimensions of Weiner et al’s [28] proposed implementation outcomes instrument, developed since, do align well with the items used in this study. As the field of mobile health (mHealth) continues to grow, instruments specifically designed to assess acceptability for this type of intervention will facilitate comparison of acceptability across individual interventions.

It is unlikely that all adolescents who fall within the intervention’s target age range will have their own smartphones (only 3 of the 60 adolescent participants in this study did at the time of the study). This intervention, if proven efficacious and made available for download, would likely be used, at least by some young adolescents, on a parent’s or other family member’s smartphone. It is very encouraging that during this study, parents appeared to find *Tumaini* highly appealing, valuable, and relevant to their children’s lives. A small number of parents’ minor concerns about the age-appropriateness of the content presented were outweighed by the majority’s endorsement of the positive changes they saw in their children and attributed to the intervention as well as the role they saw the intervention playing in preparing their children for challenges they would face.

In Kenya, primary school education has been free and compulsory since 2003, and all subjects are taught in English from standard year 4 (age 10+ years) onward, so this study’s eligibility criterion of English proficiency equivalent to grade 3 to 4 did not limit enrollment. The game’s English level, supported by an audio track, appears to have successfully matched adolescents’ proficiency, making it accessible to study participants.

The study had been scheduled to take place during the August 2017 school holiday period. However, the timeline was advanced by several months to avoid coinciding with the August 2017 Kenyan national elections. As a result, there was not enough time before the study to prepare a fully beta-tested version of *Tumaini*. This resulted in users noticing some bugs and some of the minigame mechanics proving more challenging than intended. These minor usability issues did not appear to affect the overall acceptability of the game or its appeal and, in fact, resulted in additional communication with older family members about the game as they sought help [26]. These issues will be addressed and a full bug test conducted before further game testing as originally planned.

#### Acceptability of Study

Parents had no negative experiences with or concerns about their children’s involvement in the study and indicated that they approved of the use of study-provided functionally limited phones as an intervention delivery mechanism. In addition, there was no loss to follow-up of adolescent participants, who continue to engage with the study team on additional postintervention qualitative research to this day. The only aspect of the study procedures that raised any issues were the instructions given to parents about their interactions with their children around the intervention. Although the instructions to let children play as they wished were intended to maximize adolescents’ ownership of their game experience without undue parental interference, the message was misinterpreted by a small number of parents as barring them from discussing the game with their children. In isolated cases, this resulted in less conversation and freedom of communication than anticipated. With parent-child communication, such an important protective factor for young people’s healthy decision making around sexual health [29], inadvertently minimizing discussions between adolescents and their parents about the contents of this type of intervention through misleading study instructions has the potential to limit the effects of the intervention. Before any future studies of *Tumaini*, instructions relating to parent-child communication and parents’ role in the intervention will be pretested to ensure that they are clearly understood as intended.

Based on their positive experiences with this study, parents had few reservations about a study spanning multiple years or one including the collection of blood for biomarkers. Any anticipated longitudinal efficacy trial of *Tumaini* would be planned such that study visits take place during Kenyan school holidays in line with parents’ recommendations. HIV and HSV-2 testing would follow Kenyan testing guidelines [30], which provide for pre- and posttesting counseling as well as parental presence for young adolescents, addressing parents’ concerns regarding testing procedures. In light of this, the planned efficacy study is expected to prove acceptable to parents of other adolescents in this age group. Other research from Botswana involving HSV-2 testing as a biomarker for sexual activity identified some potential barriers to acceptability from parents, which aligned with parents’ concerns in this study [31].

Gooding et al [32] note that defining trial acceptability is challenging, that it may change over the course of a study’s duration, and that it is likely to differ across participants. They encourage an early definition of trial acceptability specific to the study and its aims rather than an all-purpose definition applied to studies across the board. They additionally warn against using loss to follow-up as an indicator of continued acceptability as withdrawal, or continued participation, may result from factors external to study methods. This study was relatively short and parents’ comments on the acceptability of the planned efficacy trial may not reflect how acceptable the trial is with participants once the trial is underway. However, with these caveats, it is encouraging that participants engaged in this study do not foresee barriers to the acceptability of a longer study or one involving blood collection. Additional data collection should be carried out before and alongside the planned trial to identify, minimize, and account for emerging obstacles to continued acceptability, and not merely tolerance, of the study to all participants [33]. This study adds to the current body of literature on the development of digital interactive sexual and reproductive health interventions aimed at adolescents and young adults. It provides detailed insights into different facets of acceptability of this intervention intended to inform future trials of *Tumaini.* In addition, it discusses the importance of assessing the acceptability of an intervention and of study methods before undertaking large-scale efficacy trials and the value of developing standards to allow for the comparison of interventions in terms of acceptability.

### Limitations

This study included 30 adolescents in the intervention group and 27 adolescents and 22 parents in the FGDs, and the intervention period was just over 2 weeks. As most of the parents were female, it is possible that fathers may have had a different perception of the game and study. Adolescents were not asked about the acceptability of blood tests or of a longitudinal study, and acceptability of a future study was based on a hypothetical description of a study rather than a detailed study plan. This study was conducted in Kisumu, western Kenya, and the findings may not be generalizable throughout Kenya or to other parts of East Africa.

### Conclusions

As researchers develop innovative mHealth prevention interventions and strengthen efficacy testing, they must consider the acceptability of the intervention and trial as crucial to success. For interventions aimed at adolescents, acceptability must include acceptability to parents to minimize barriers to trial retention and intervention uptake. Our study findings show a high level of acceptability for a smartphone-based HIV prevention serious game intervention among adolescents and their parents as well as the methods used to conduct the study. The acceptability constructs used in our study align with new frameworks proposed by Weiner [28] and Gooding [32], and we hope they can help inform similar intervention studies.

## Abbreviations

FGD: focus group discussion
HSV-2: herpes simplex virus type 2
KEMRI: Kenya Medical Research Institute
mHealth: mobile health
TAM: Technology Acceptance Model

## Appendix

Multimedia Appendix 1 Sample Tumaini Graphics.

Multimedia Appendix 2 CONSORT-EHEALTH checklist (V 1.6.1).
