# Supplementary material for: A Smartphone Game to Prevent HIV Among Young Africans (Tumaini): Assessing Intervention and Study Acceptability Among Adolescents and Their Parents in a Randomized Controlled Trial
Source: JMIR Mhealth Uhealth. 2019 May 21;7(5):e13049. doi: 10.2196/13049 (PMC6547768; doi:10.2196/13049)

**Multimedia Appendix 1.**

**Sample *Tumaini* graphics**

Sample graphic from narrative


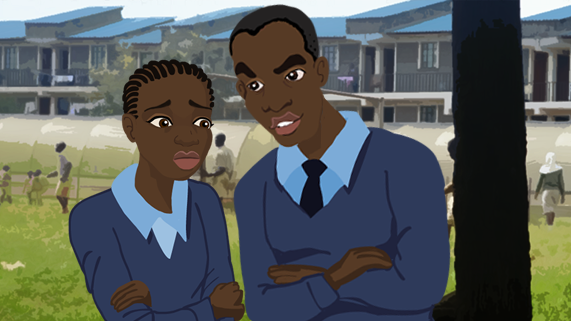


Sample graphic from mini-games


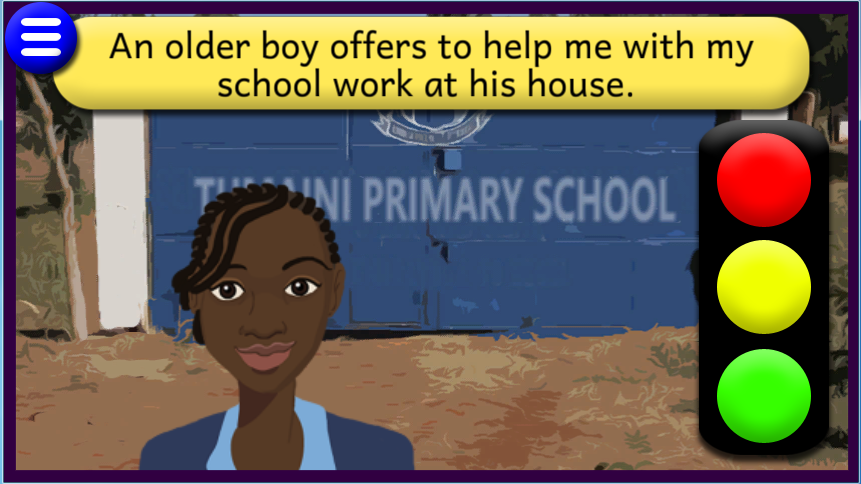


Sample graphic from My Story


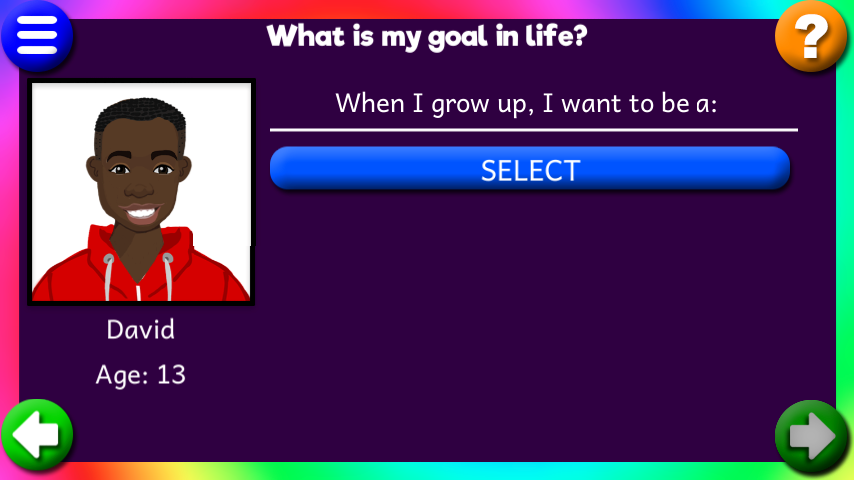

Supplement: Multimedia Appendix 1 [file mhealth_v7i5e13049_app1.docx]
