# Supplementary material for: A Smartphone Game to Prevent HIV Among Young Africans (Tumaini): Assessing Intervention and Study Acceptability Among Adolescents and Their Parents in a Randomized Controlled Trial
Source: JMIR Mhealth Uhealth. 2019 May 21;7(5):e13049. doi: 10.2196/13049 (PMC6547768; doi:10.2196/13049)
Supplement: Multimedia Appendix 2 [file mhealth_v7i5e13049_app2.pdf]

CONSORT-EHEALTH (V 1.6.1) - Submission/Publication Form

The CONSORT-EHEALTH checklist is intended for authors of randomized trials evaluating web-based and Internet-based applications/interventions, including mobile interventions, electronic games (incl multiplayer games), social media, certain telehealth applications, and other interactive and/or networked electronic applications. Some of the items (e.g. all subitems under item 5 - description of the intervention) may also be applicable for other study designs.

The goal of the CONSORT EHEALTH checklist and guideline is to be
a) a guide for reporting for authors of RCTs,
b) to form a basis for appraisal of an ehealth trial (in terms of validity)

CONSORT-EHEALTH items/subitems are MANDATORY reporting items for studies published in the Journal of Medical Internet Research and other journals / scientific societies endorsing the checklist.

Items numbered 1., 2., 3., 4a., 4b etc are original CONSORT or CONSORT-NPT (non-pharmacologic treatment) items.
Items with Roman numerals (i., ii, iii, iv etc.) are CONSORT-EHEALTH extensions/clarifications.

As the CONSORT-EHEALTH checklist is still considered in a formative stage, we would ask that you also RATE ON A SCALE OF 1-5 how important/useful you feel each item is FOR THE PURPOSE OF THE CHECKLIST and reporting guideline (optional).

Mandatory reporting items are marked with a red *.
In the textboxes, either copy & paste the relevant sections from your manuscript into this form - please include any quotes from your manuscript in QUOTATION MARKS,
or answer directly by providing additional information not in the manuscript, or elaborating on why the item was not relevant for this study.

YOUR ANSWERS WILL BE PUBLISHED AS A SUPPLEMENTARY FILE TO YOUR PUBLICATION IN JMIR AND ARE CONSIDERED PART OF YOUR PUBLICATION (IF ACCEPTED).
Please fill in these questions diligently. Information will not be copyedited, so please use proper spelling and grammar, use correct capitalization, and avoid abbreviations.

DO NOT FORGET TO SAVE AS PDF _AND_ CLICK THE SUBMIT BUTTON SO YOUR ANSWERS ARE IN OUR DATABASE !!!

Citation Suggestion (if you append the pdf as Appendix we suggest to cite this paper in the caption):
Eysenbach G, CONSORT-EHEALTH Group
CONSORT-EHEALTH: Improving and Standardizing Evaluation Reports of Web-based and Mobile Health Interventions
J Med Internet Res 2011;13(4):e126
URL: http://www.jmir.org/2011/4/e126/
doi: 10.2196/jmir.1923
PMID: 22209829

* Required

Your name *

First Last

Kate Winskell

Primary Affiliation (short), City, Country *

University of Toronto, Toronto, Canada

Emory University, Atlanta, USA

Your e-mail address *

abc@gmail.com

swinske@emory.edu

Title of your manuscript *

Provide the (draft) title of your manuscript.

A Smartphone Game-Based Intervention to Prevent HIV Among Young Africans (Tumaini): Pilot Randomized Controlled Trial

Name of your App/Software/Intervention *

If there is a short and a long/alternate name, write the short name first and add the long name in brackets.

Tumaini

Evaluated Version (if any)

e.g. "V1", "Release 2017-03-01", "Version 2.0.27913"

Your answer

Language(s) *

What language is the intervention/app in? If multiple languages are available, separate by comma (e.g. "English, French")

English

URL of your Intervention Website or App

e.g. a direct link to the mobile app on app in appstore (itunes, Google Play), or URL of the website. If the intervention is a DVD or hardware, you can also link to an Amazon page.

Your answer

URL of an image/screenshot (optional)

Your answer

Accessibility *

Can an enduser access the intervention presently?

- ○ access is free and open
- ◉ access only for special usergroups, not open
- ○ access is open to everyone, but requires payment/subscription/in-app purchases
- ○ app/intervention no longer accessible
- ○ Other:

Primary Medical Indication/Disease/Condition *

e.g. "Stress", "Diabetes", or define the target group in brackets after the condition, e.g. "Autism (Parents of children with)", "Alzheimers (Informal Caregivers of)"

HIV, sexual and reproductive health

Primary Outcomes measured in trial *

comma-separated list of primary outcomes reported in the trial

Recruitment rate, Time to recruitment of

Secondary/other outcomes

Are there any other outcomes the intervention is expected to affect?

Change in propositional knowledge, change in procedural knowledge, change in self-efficacy, change in perceived risk, change in behavioral intention, change in future orientation, change in perceived social norms

Recommended "Dose" *

What do the instructions for users say on how often the app should be used?

- ( ) Approximately Daily
- ( ) Approximately Weekly
- ( ) Approximately Monthly
- ( ) Approximately Yearly
- (x) "as needed"
- ( ) Other:

Approx. Percentage of Users (starters) still using the app as recommended after 3 months *

- (x) unknown / not evaluated
- ( ) 0-10%
- ( ) 11-20%
- ( ) 21-30%
- ( ) 31-40%
- ( ) 41-50%
- ( ) 51-60%
- ( ) 61-70%
- ( ) 71%-80%
- ( ) 81-90%
- ( ) 91-100%
- ( ) Other:

Overall, was the app/intervention effective? *

- ( ) yes: all primary outcomes were significantly better in intervention group vs control
- ( ) partly: SOME primary outcomes were significantly better in intervention group vs control
- ( ) no statistically significant difference between control and intervention
- ( ) potentially harmful: control was significantly better than intervention in one or more outcomes
- ( ) inconclusive: more research is needed
- (x) Other: This is a feasibility study that was not powered to detect efficacy' prima

Article Preparation Status/Stage *

At which stage in your article preparation are you currently (at the time you fill in this form)

- ○ not submitted yet - in early draft status
- ○ not submitted yet - in late draft status, just before submission
- ○ submitted to a journal but not reviewed yet
- ○ submitted to a journal and after receiving initial reviewer comments
- ◉ submitted to a journal and accepted, but not published yet
- ○ published
- ○ Other:

Journal *

If you already know where you will submit this paper (or if it is already submitted), please provide the journal name (if it is not JMIR, provide the journal name under "other")

- ○ not submitted yet / unclear where I will submit this
- ○ Journal of Medical Internet Research (JMIR)
- ◉ JMIR mHealth and UHealth
- ○ JMIR Serious Games
- ○ JMIR Mental Health
- ○ JMIR Public Health
- ○ JMIR Formative Research
- ○ Other JMIR sister journal
- ○ Other:

Is this a full powered effectiveness trial or a pilot/feasibility trial? *

- ◉ Pilot/feasibility
- ○ Fully powered

Manuscript tracking number *

If this is a JMIR submission, please provide the manuscript tracking number under "other" (The ms tracking number can be found in the submission acknowledgement email, or when you login as author in JMIR. If the paper is already published in JMIR, then the ms tracking number is the four-digit number at the end of the DOI, to be found at the bottom of each published article in JMIR)

- ○ no ms number (yet) / not (yet) submitted to / published in JMIR
- ◉ Other: 10482

TITLE AND ABSTRACT

1a) TITLE: Identification as a randomized trial in the title

1a) Does your paper address CONSORT item 1a? *

I.e does the title contain the phrase "Randomized Controlled Trial"? (if not, explain the reason under "other")

- ◉ yes
- ○ Other:

1a-i) Identify the mode of delivery in the title

Identify the mode of delivery. Preferably use “web-based” and/or “mobile” and/or “electronic game” in the title. Avoid ambiguous terms like “online”, “virtual”, “interactive”. Use “Internet-based” only if Intervention includes non-web-based Internet components (e.g. email), use “computer-based” or “electronic” only if offline products are used. Use “virtual” only in the context of “virtual reality” (3-D worlds). Use “online” only in the context of “online support groups”. Complement or substitute product names with broader terms for the class of products (such as “mobile” or “smart phone” instead of “iphone”), especially if the application runs on different platforms.

	1	2	3	4	5	
subitem not at all important	○	○	○	○	○	essential

Does your paper address subitem 1a-i? *

Copy and paste relevant sections from manuscript title (include quotes in quotation marks "like this" to indicate direct quotes from your manuscript), or elaborate on this item by providing additional information not in the ms, or briefly explain why the item is not applicable/relevant for your study

"A Smartphone Game-Based Intervention"

1a-ii) Non-web-based components or important co-interventions in title

Mention non-web-based components or important co-interventions in title, if any (e.g., "with telephone support").

	1	2	3	4	5	
subitem not at all important	○	○	○	○	○	essential

Does your paper address subitem 1a-ii?

Copy and paste relevant sections from manuscript title (include quotes in quotation marks "like this" to indicate direct quotes from your manuscript), or elaborate on this item by providing additional information not in the ms, or briefly explain why the item is not applicable/relevant for your study

There were no other co-interventions or non-smartphone support

1a-iii) Primary condition or target group in the title

Mention primary condition or target group in the title, if any (e.g., "for children with Type I Diabetes") Example: A Web-based and Mobile Intervention with Telephone Support for Children with Type I Diabetes: Randomized Controlled Trial

	1	2	3	4	5	
subitem not at all important	○	○	○	○	○	essential

Does your paper address subitem 1a-iii? *

Copy and paste relevant sections from manuscript title (include quotes in quotation marks "like this" to indicate direct quotes from your manuscript), or elaborate on this item by providing additional information not in the ms, or briefly explain why the item is not applicable/relevant for your study

"to Prevent HIV Among Young Africans"

1b) ABSTRACT: Structured summary of trial design, methods, results, and conclusions

NPT extension: Description of experimental treatment, comparator, care providers, centers, and blinding status.

1b-i) Key features/functionalities/components of the intervention and comparator in the METHODS section of the ABSTRACT

Mention key features/functionalities/components of the intervention and comparator in the abstract. If possible, also mention theories and principles used for designing the site. Keep in mind the needs of systematic reviewers and indexers by including important synonyms. (Note: Only report in the abstract what the main paper is reporting. If this information is missing from the main body of text, consider adding it)

	1	2	3	4	5	
subitem not at all important	○	○	○	○	○	essential

Does your paper address subitem 1b-i? *

Copy and paste relevant sections from the manuscript abstract (include quotes in quotation marks "like this" to indicate direct quotes from your manuscript), or elaborate on this item by providing additional information not in the ms, or briefly explain why the item is not applicable/relevant for your study

Tumaini (“hope for the future” in Swahili) is an interactive, narrative-based game grounded in social cognitive theory. Intervention arm participants (n=30) were provided with an Android smartphone with Tumaini installed on it and were instructed to play the game for at least 1 hour a day for 16 days; control arm participants (n=30) received no intervention.

1b-ii) Level of human involvement in the METHODS section of the ABSTRACT

Clarify the level of human involvement in the abstract, e.g., use phrases like “fully automated” vs. “therapist/nurse/care provider/physician-assisted” (mention number and expertise of providers involved, if any). (Note: Only report in the abstract what the main paper is reporting. If this information is missing from the main body of text, consider adding it)

	1	2	3	4	5	
subitem not at all important	○	○	○	○	○	essential

Does your paper address subitem 1b-ii?

Copy and paste relevant sections from the manuscript abstract (include quotes in quotation marks "like this" to indicate direct quotes from your manuscript), or elaborate on this item by providing additional information not in the ms, or briefly explain why the item is not applicable/relevant for your study

"Intervention arm participants (n=30) were provided with an Android smartphone with Tumaini installed on it and were instructed to play the game for at least 1 hour a day for 16 days" - As implied here, there was no human involvement related to the intervention once participants received the devices. Participants could reach out to study staff if questions arose.

1b-iii) Open vs. closed, web-based (self-assessment) vs. face-to-face assessments in the METHODS section of the ABSTRACT

Mention how participants were recruited (online vs. offline), e.g., from an open access website or from a clinic or a closed online user group (closed usergroup trial), and clarify if this was a purely web-based trial, or there were face-to-face components (as part of the intervention or for assessment). Clearly say if outcomes were self-assessed through questionnaires (as common in web-based trials). Note: In traditional offline trials, an open trial (open-label trial) is a type of clinical trial in which both the researchers and participants know which treatment is being administered. To avoid confusion, use “blinded” or “unblinded” to indicated the level of blinding instead of “open”, as “open” in web-based trials usually refers to “open access” (i.e. participants can self-enrol). (Note: Only report in the abstract what the main paper is reporting. If this information is missing from the main body of text, consider adding it)

	1	2	3	4	5	
subitem not at all important	○	○	○	○	○	essential

Does your paper address subitem 1b-iii?

Copy and paste relevant sections from the manuscript abstract (include quotes in quotation marks "like this" to indicate direct quotes from your manuscript), or elaborate on this item by providing additional information not in the ms, or briefly explain why the item is not applicable/relevant for your study

"All participants completed a survey on behavioral mediators, delivered via an audio computer-assisted self-interview system at baseline (T1), post intervention (T2), and at 6 weeks postintervention (T3)." "Intervention arm participants and their parents participated in 8 postintervention focus group discussions." Participants were recruited through school-initiated contact via letters given to eligible children inviting parents to attend informational sessions.

1b-iv) RESULTS section in abstract must contain use data

Report number of participants enrolled/assessed in each group, the use/uptake of the intervention (e.g., attrition/adherence metrics, use over time, number of logins etc.), in addition to primary/secondary outcomes. (Note: Only report in the abstract what the main paper is reporting. If this information is missing from the main body of text, consider adding it)

	1	2	3	4	5	
subitem not at all important	○	○	○	○	○	essential

Does your paper address subitem 1b-iv?

Copy and paste relevant sections from the manuscript abstract (include quotes in quotation marks "like this" to indicate direct quotes from your manuscript), or elaborate on this item by providing additional information not in the ms, or briefly explain why the item is not applicable/relevant for your study

Number of participants is included in the methods: "with 60 participants aged 11-14 (mean 12.7) years. Intervention arm participants (n=30)... control arm participants (n=30)"
"Intervention arm participants played Tumaini for a mean of approximately 27 hours."

1b-v) CONCLUSIONS/DISCUSSION in abstract for negative trials

Conclusions/Discussions in abstract for negative trials: Discuss the primary outcome - if the trial is negative (primary outcome not changed), and the intervention was not used, discuss whether negative results are attributable to lack of uptake and discuss reasons. (Note: Only report in the abstract what the main paper is reporting. If this information is missing from the main body of text, consider adding it)

	1	2	3	4	5	
subitem not at all important	○	○	○	○	○	essential

Does your paper address subitem 1b-v?

Copy and paste relevant sections from the manuscript abstract (include quotes in quotation marks "like this" to indicate direct quotes from your manuscript), or elaborate on this item by providing additional information not in the ms, or briefly explain why the item is not applicable/relevant for your study

The trial had significant findings.

INTRODUCTION

2a) In INTRODUCTION: Scientific background and explanation of rationale

2a-i) Problem and the type of system/solution

Describe the problem and the type of system/solution that is object of the study: intended as stand-alone intervention vs. incorporated in broader health care program? Intended for a particular patient population? Goals of the intervention, e.g., being more cost-effective to other interventions, replace or complement other solutions? (Note: Details about the intervention are provided in "Methods" under 5)

	1	2	3	4	5	
subitem not at all important	○	○	○	○	○	essential

Does your paper address subitem 2a-i? *

Copy and paste relevant sections from the manuscript (include quotes in quotation marks "like this" to indicate direct quotes from your manuscript), or elaborate on this item by providing additional information not in the ms, or briefly explain why the item is not applicable/relevant for your study

"A third of all new adult HIV infections occur in young people aged 15-24 years [1]. In African countries most affected by HIV, demographic change is increasing the size of adolescent cohorts, thereby increasing their contribution to HIV incidence [2]. In addition, this age group suffers disproportionately high levels of HIV-related morbidity and mortality [3]. Reaching preadolescents with prerisk prevention interventions may help establish lifelong patterns of safer sexual behavior and avert high-risk behaviors in the future" “In addition to their appeal, games for sexual health have further distinctive advantages over common group-based evidence-based interventions [29]. They have considerable potential for scalability, low cost per person reached, and cultural adaptability. Exposure to the intervention can be reliably measured through automated data-collection, which can also help pinpoint “active ingredients,” contributing to the building of behavioral, pedagogical, and game design theory. Fidelity to intervention design is much more likely as the intervention is no longer dependent on a skilled cadre of facilitators. Electronic delivery offers potential for remote updates, while portability via mobile handsets can allow the intervention to link into people’s everyday lives, offering more sustained intervention exposure.” "There is a pressing need to assess the feasibility of using game technologies for HIV prevention in low-resource settings and their potential for efficacy". The manuscript describes a feasibility study in which a smartphone-based game was used as a stand-alone intervention. The piloted game models dialogue with trusted adults and access to services

2a-ii) Scientific background, rationale: What is known about the (type of) system

Scientific background, rationale: What is known about the (type of) system that is the object of the study (be sure to discuss the use of similar systems for other conditions/diagnoses, if appropiate), motivation for the study, i.e. what are the reasons for and what is the context for this specific study, from which stakeholder viewpoint is the study performed, potential impact of findings [2]. Briefly justify the choice of the comparator.

	1	2	3	4	5	
subitem not at all important	○	○	○	○	○	essential

Does your paper address subitem 2a-ii? *

Copy and paste relevant sections from the manuscript (include quotes in quotation marks "like this" to indicate direct quotes from your manuscript), or elaborate on this item by providing additional information not in the ms, or briefly explain why the item is not applicable/relevant for your study

"Electronic games have the potential to be a valuable tool in youth HIV prevention in Sub-Saharan Africa if they are appropriately grounded in behavioral and instructional theory [8,9], informed by existing evidence-based interventions [10], and contextually appropriate. Smartphone ownership is increasing dramatically in emerging and developing nations [11], opening up new possibilities for delivering highly interactive, culturally relevant mHealth interventions at scale and low cost. Serious digital games [12] have high entertainment and motivational appeal for young people. They also have distinctive advantages from the perspective of pedagogy and behavioral theory. By allowing players to experience real agency in a virtual and safe environment, well-designed games provide a level of experiential learning unparalleled by many other interventions. They are particularly well aligned with key constructs of social cognitive theory [13], allowing for both cognitive and behavioral rehearsal through role-play and simulation. Although a relatively limited number of games to date have been designed with solid theoretical grounding and rigorously evaluated [14-20], there is evidence of their effectiveness for health, including clinical outcomes [21-28]." The choice of comparator (no intervention) is justified in the Methods section: it reflects current standard of care.

2b) In INTRODUCTION: Specific objectives or hypotheses

Does your paper address CONSORT subitem 2b? *

Copy and paste relevant sections from the manuscript (include quotes in quotation marks "like this" to indicate direct quotes from your manuscript), or elaborate on this item by providing additional information not in the ms, or briefly explain why the item is not applicable/relevant for your study

"There is a pressing need to assess the feasibility of using game technologies for HIV prevention in low-resource settings and their potential for efficacy. In this study, we pilot-tested an interactive narrative-based smartphone game to prevent HIV among preadolescents in Kisumu Town, Western Kenya, where adult HIV prevalence (19.9%) is over three times the national average [30,31]. We describe here results from this pilot study of the game's potential to influence behavioral mediators of increased age and condom use at sexual debut."

METHODS

3a) Description of trial design (such as parallel, factorial) including allocation ratio

Does your paper address CONSORT subitem 3a? *

Copy and paste relevant sections from the manuscript (include quotes in quotation marks "like this" to indicate direct quotes from your manuscript), or elaborate on this item by providing additional information not in the ms, or briefly explain why the item is not applicable/relevant for your study

"We conducted an individually randomized pilot study of the game Tumaini" "Participants (n=60) were randomized 1:1 to the control arm (n=30) or the intervention (game) arm (n=30) of the study."

3b) Important changes to methods after trial commencement (such as eligibility criteria), with reasons

Does your paper address CONSORT subitem 3b? *

Copy and paste relevant sections from the manuscript (include quotes in quotation marks "like this" to indicate direct quotes from your manuscript), or elaborate on this item by providing additional information not in the ms, or briefly explain why the item is not applicable/relevant for your study

There were no changes after trial commencement.

3b-i) Bug fixes, Downtimes, Content Changes

Bug fixes, Downtimes, Content Changes: ehealth systems are often dynamic systems. A description of changes to methods therefore also includes important changes made on the intervention or comparator during the trial (e.g., major bug fixes or changes in the functionality or content) (5-iii) and other “unexpected events” that may have influenced study design such as staff changes, system failures/downtimes, etc. [2].

	1	2	3	4	5	
subitem not at all important	○	○	○	○	○	essential

Does your paper address subitem 3b-i?

Copy and paste relevant sections from the manuscript (include quotes in quotation marks "like this" to indicate direct quotes from your manuscript), or elaborate on this item by providing additional information not in the ms, or briefly explain why the item is not applicable/relevant for your study

There were no changes after study commencement.

4a) Eligibility criteria for participants

Does your paper address CONSORT subitem 4a? *

Copy and paste relevant sections from the manuscript (include quotes in quotation marks "like this" to indicate direct quotes from your manuscript), or elaborate on this item by providing additional information not in the ms, or briefly explain why the item is not applicable/relevant for your study

"The eligibility criteria for participation were as follows: age 11-14 years, grade 3-4 English proficiency on the Flesch-Kincaid Reading Scale, residence in Kisumu Town, and willingness to complete all study activities."

4a-i) Computer / Internet literacy

Computer / Internet literacy is often an implicit “de facto” eligibility criterion - this should be explicitly clarified.

	1	2	3	4	5	
subitem not at all important	○	○	○	○	○	essential

Does your paper address subitem 4a-i?

Copy and paste relevant sections from the manuscript (include quotes in quotation marks "like this" to indicate direct quotes from your manuscript), or elaborate on this item by providing additional information not in the ms, or briefly explain why the item is not applicable/relevant for your study

This was a feasibility study.

4a-ii) Open vs. closed, web-based vs. face-to-face assessments:

Open vs. closed, web-based vs. face-to-face assessments: Mention how participants were recruited (online vs. offline), e.g., from an open access website or from a clinic, and clarify if this was a purely web-based trial, or there were face-to-face components (as part of the intervention or for assessment), i.e., to what degree got the study team to know the participant. In online-only trials, clarify if participants were quasi-anonymous and whether having multiple identities was possible or whether technical or logistical measures (e.g., cookies, email confirmation, phone calls) were used to detect/prevent these.

	1	2	3	4	5	
subitem not at all important	○	○	○	○	○	essential

Does your paper address subitem 4a-ii? *

Copy and paste relevant sections from the manuscript (include quotes in quotation marks "like this" to indicate direct quotes from your manuscript), or elaborate on this item by providing additional information not in the ms, or briefly explain why the item is not applicable/relevant for your study

"Letters were distributed through schools to parents of age-eligible children inviting them to attend informational meetings."
"All participants completed a self-administered behavioral survey at T1, T2, and T3. The English language survey was completed at the KEMRI offices, using the audio computer-assisted self-interview (ACASI) system with headphones to protect privacy. It took approximately 1 hour to complete."
"Intervention arm participants (n=27) and their parents (n=22) took part in FGDs (n=8) between T2 and T3."
"Data from the phone log files were downloaded as .txt files and converted into Excel files,"

4a-iii) Information giving during recruitment

Information given during recruitment. Specify how participants were briefed for recruitment and in the informed consent procedures (e.g., publish the informed consent documentation as appendix, see also item X26), as this information may have an effect on user self-selection, user expectation and may also bias results.

	1	2	3	4	5	
subitem not at all important	○	○	○	○	○	essential

Does your paper address subitem 4a-iii?

Copy and paste relevant sections from the manuscript (include quotes in quotation marks "like this" to indicate direct quotes from your manuscript), or elaborate on this item by providing additional information not in the ms, or briefly explain why the item is not applicable/relevant for your study

"Letters were distributed through schools to parents of age-eligible children inviting them to attend informational meetings. Consent and assent were secured at the home of the participants, following an explanation of the study. Parents consented to participate in the postintervention focus groups if their child was randomized to the intervention arm." Informational meetings included general information about the potential of the game and the problems it aims to address as well as a description of the study. Consent procedures reviewed this content.
"Assignments were revealed to participants after they had completed the baseline assessment"
"Intervention arm participants completed a 45-minute informational onboarding session, including instructions on the interface, technology, and game content."
Parents of intervention arm participants also attended an onboarding session and received the same information as their children about the game's content.

4b) Settings and locations where the data were collected

Does your paper address CONSORT subitem 4b? *

Copy and paste relevant sections from the manuscript (include quotes in quotation marks "like this" to indicate direct quotes from your manuscript), or elaborate on this item by providing additional information not in the ms, or briefly explain why the item is not applicable/relevant for your study

"The English language survey was completed at the KEMRI offices, using the audio computer-assisted self-interview (ACASI) system with headphones to protect privacy." The manuscript indicates that participants also took part in FGDs- these were carried out at the KEMRI offices and in the offices of local CBOs.

4b-i) Report if outcomes were (self-)assessed through online questionnaires

Clearly report if outcomes were (self-)assessed through online questionnaires (as common in web-based trials) or otherwise.

	1	2	3	4	5	
subitem not at all important	○	○	○	○	○	essential

Does your paper address subitem 4b-i? *

Copy and paste relevant sections from the manuscript (include quotes in quotation marks "like this" to indicate direct quotes from your manuscript), or elaborate on this item by providing additional information not in the ms, or briefly explain why the item is not applicable/relevant for your study

The survey was carried out via ACASI in person. Data was also downloaded from the study phones. There was no online questionnaire component.

4b-ii) Report how institutional affiliations are displayed

Report how institutional affiliations are displayed to potential participants [on ehealth media], as affiliations with prestigious hospitals or universities may affect volunteer rates, use, and reactions with regards to an intervention.(Not a required item – describe only if this may bias results)

	1	2	3	4	5	
subitem not at all important	○	○	○	○	○	essential

Does your paper address subitem 4b-ii?

Copy and paste relevant sections from the manuscript (include quotes in quotation marks "like this" to indicate direct quotes from your manuscript), or elaborate on this item by providing additional information not in the ms, or briefly explain why the item is not applicable/relevant for your study

The participants were told about institutional affiliations during recruitment and consenting, and study activities occurred at the KEMRI offices.

5) The interventions for each group with sufficient details to allow replication, including how and when they were actually administered

5-i) Mention names, credential, affiliations of the developers, sponsors, and owners

Mention names, credential, affiliations of the developers, sponsors, and owners [6] (if authors/evaluators are owners or developer of the software, this needs to be declared in a "Conflict of interest" section or mentioned elsewhere in the manuscript).

	1	2	3	4	5	
subitem not at all important	○	○	○	○	○	essential

Does your paper address subitem 5-i?

Copy and paste relevant sections from the manuscript (include quotes in quotation marks "like this" to indicate direct quotes from your manuscript), or elaborate on this item by providing additional information not in the ms, or briefly explain why the item is not applicable/relevant for your study

"game for inexpensive Android smartphones developed in collaboration with a US commercial game developer, Realtime Associates, and with input from US-based and Kenyan specialists in adolescent sexual health and Kenyan preadolescents and their parents."
"Research reported in this publication was supported by the National Institute of Mental Health of the US National Institutes of Health under Award Number 5R34MH106368 (PI: KW)."

5-ii) Describe the history/development process

Describe the history/development process of the application and previous formative evaluations (e.g., focus groups, usability testing), as these will have an impact on adoption/use rates and help with interpreting results.

	1	2	3	4	5	
subitem not at all important	○	○	○	○	○	essential

Does your paper address subitem 5-ii?

Copy and paste relevant sections from the manuscript (include quotes in quotation marks "like this" to indicate direct quotes from your manuscript), or elaborate on this item by providing additional information not in the ms, or briefly explain why the item is not applicable/relevant for your study

This is a feasibility study so it is part of the development process. Additional information included in this paper: "developed in collaboration with a US commercial game developer, Realtime Associates, and with input from US-based and Kenyan specialists in adolescent sexual health and Kenyan preadolescents and their parents."

5-iii) Revisions and updating

Revisions and updating. Clearly mention the date and/or version number of the application/intervention (and comparator, if applicable) evaluated, or describe whether the intervention underwent major changes during the evaluation process, or whether the development and/or content was “frozen” during the trial. Describe dynamic components such as news feeds or changing content which may have an impact on the replicability of the intervention (for unexpected events see item 3b).

	1	2	3	4	5	
subitem not at all important	○	○	○	○	○	essential

Does your paper address subitem 5-iii?

Copy and paste relevant sections from the manuscript (include quotes in quotation marks "like this" to indicate direct quotes from your manuscript), or elaborate on this item by providing additional information not in the ms, or briefly explain why the item is not applicable/relevant for your study

There were no revisions or updates during the trial

5-iv) Quality assurance methods

Provide information on quality assurance methods to ensure accuracy and quality of information provided [1], if applicable.

	1	2	3	4	5	
subitem not at all important	○	○	○	○	○	essential

Does your paper address subitem 5-iv?

Copy and paste relevant sections from the manuscript (include quotes in quotation marks "like this" to indicate direct quotes from your manuscript), or elaborate on this item by providing additional information not in the ms, or briefly explain why the item is not applicable/relevant for your study

The game was developed "with input from US-based and Kenyan specialists in adolescent sexual health"

5-v) Ensure replicability by publishing the source code, and/or providing screenshots/screen-capture video, and/or providing flowcharts of the algorithms used

Ensure replicability by publishing the source code, and/or providing screenshots/screen-capture video, and/or providing flowcharts of the algorithms used. Replicability (i.e., other researchers should in principle be able to replicate the study) is a hallmark of scientific reporting.

	1	2	3	4	5	
subitem not at all important	○	○	○	○	○	essential

Does your paper address subitem 5-v?

Copy and paste relevant sections from the manuscript (include quotes in quotation marks "like this" to indicate direct quotes from your manuscript), or elaborate on this item by providing additional information not in the ms, or briefly explain why the item is not applicable/relevant for your study

We provide illustrative screenshots.

5-vi) Digital preservation

Digital preservation: Provide the URL of the application, but as the intervention is likely to change or disappear over the course of the years; also make sure the intervention is archived (Internet Archive, webcitation.org, and/or publishing the source code or screenshots/videos alongside the article). As pages behind login screens cannot be archived, consider creating demo pages which are accessible without login.

	1	2	3	4	5	
subitem not at all important	○	○	○	○	○	essential

Does your paper address subitem 5-vi?

Copy and paste relevant sections from the manuscript (include quotes in quotation marks "like this" to indicate direct quotes from your manuscript), or elaborate on this item by providing additional information not in the ms, or briefly explain why the item is not applicable/relevant for your study

The app will eventually be made available if proven efficacious. At this stage, it is not online nor widely accessible. Only study participants are provided with the intervention.

5-vii) Access

Access: Describe how participants accessed the application, in what setting/context, if they had to pay (or were paid) or not, whether they had to be a member of specific group. If known, describe how participants obtained "access to the platform and Internet" [1]. To ensure access for editors/reviewers/readers, consider to provide a "backdoor" login account or demo mode for reviewers/readers to explore the application (also important for archiving purposes, see vi).

	1	2	3	4	5	
subitem not at all important	○	○	○	○	○	essential

Does your paper address subitem 5-vii? *

Copy and paste relevant sections from the manuscript (include quotes in quotation marks "like this" to indicate direct quotes from your manuscript), or elaborate on this item by providing additional information not in the ms, or briefly explain why the item is not applicable/relevant for your study

Intervention participants were provided with a phone with
the game preloaded and used it at their own pace for the duration
of the intervention.

5-viii) Mode of delivery, features/functionalities/components of the intervention and comparator, and the theoretical framework

Describe mode of delivery, features/functionalities/components of the intervention and comparator, and the theoretical framework [6] used to design them (instructional strategy [1], behaviour change techniques, persuasive features, etc., see e.g., [7, 8] for terminology). This includes an in-depth description of the content (including where it is coming from and who developed it) [1]," whether [and how] it is tailored to individual circumstances and allows users to track their progress and receive feedback" [6]. This also includes a description of communication delivery channels and – if computer-mediated communication is a component – whether communication was synchronous or asynchronous [6]. It also includes information on presentation strategies [1], including page design principles, average amount of text on pages, presence of hyperlinks to other resources, etc. [1].

	1	2	3	4	5	
subitem not at all important	○	○	○	○	○	essential

Does your paper address subitem 5-viii? *

Copy and paste relevant sections from the manuscript (include quotes in quotation marks "like this" to indicate direct quotes from your manuscript), or elaborate on this item by providing additional information not in the ms, or briefly explain why the item is not applicable/relevant for your study

The game is "a theoretically grounded,
narrative-based game for inexpensive Android smartphones
developed in collaboration with a US commercial game
developer, Realtime Associates, and with input from US-based
and Kenyan specialists in adolescent sexual health and Kenyan
preadolescents and their parents."
"The game's design draws on social behavioral theory, including
social cognitive theory [13] and the theory of possible selves
[32]; existing evidence-based interventions for youth HIV
prevention [33,34-36]; and games for health [8,12,17,37] and
entertainment-education [38] literature. It is grounded in research
on HIV-themed narratives written by young Africans [39-41].
Tumaini uses interactive narrative to promote observational
learning, cognitive and behavioral rehearsal, problem-solving,
and immersion."
The game is made up of 3 intersecting components: (1) a role-playing narrative;
(2) a set of mini-games; (3) a self-reflection component. The topics of (2) and (3)
are tied to the narrative.

5-ix) Describe use parameters

Describe use parameters (e.g., intended "doses" and optimal timing for use). Clarify what instructions or recommendations were given to the user, e.g., regarding timing, frequency, heaviness of use, if any, or was the intervention used ad libitum.

	1	2	3	4	5	
subitem not at all important	○	○	○	○	○	essential

Does your paper address subitem 5-ix?

Copy and paste relevant sections from the manuscript (include quotes in quotation marks "like this" to indicate direct quotes from your manuscript), or elaborate on this item by providing additional information not in the ms, or briefly explain why the item is not applicable/relevant for your study

"They were instructed to play at least 1 hour per day for the 16 days of the study and asked not to share their own gameplay profile with others." Participants "used it at their own pace for the duration of the intervention."

5-x) Clarify the level of human involvement

Clarify the level of human involvement (care providers or health professionals, also technical assistance) in the e-intervention or as co-intervention (detail number and expertise of professionals involved, if any, as well as "type of assistance offered, the timing and frequency of the support, how it is initiated, and the medium by which the assistance is delivered". It may be necessary to distinguish between the level of human involvement required for the trial, and the level of human involvement required for a routine application outside of a RCT setting (discuss under item 21 – generalizability).

	1	2	3	4	5	
subitem not at all important	○	○	○	○	○	essential

Does your paper address subitem 5-x?

Copy and paste relevant sections from the manuscript (include quotes in quotation marks "like this" to indicate direct quotes from your manuscript), or elaborate on this item by providing additional information not in the ms, or briefly explain why the item is not applicable/relevant for your study

Participants and their parents were told to reach out to study staff with any questions that arose as needed.

5-xi) Report any prompts/reminders used

Report any prompts/reminders used: Clarify if there were prompts (letters, emails, phone calls, SMS) to use the application, what triggered them, frequency etc. It may be necessary to distinguish between the level of prompts/reminders required for the trial, and the level of prompts/reminders for a routine application outside of a RCT setting (discuss under item 21 – generalizability).

	1	2	3	4	5	
subitem not at all important	○	○	○	○	○	essential

Does your paper address subitem 5-xi? *

Copy and paste relevant sections from the manuscript (include quotes in quotation marks "like this" to indicate direct quotes from your manuscript), or elaborate on this item by providing additional information not in the ms, or briefly explain why the item is not applicable/relevant for your study

An alarm set to ring once a day reminded players to play.

5-xii) Describe any co-interventions (incl. training/support)

Describe any co-interventions (incl. training/support): Clearly state any interventions that are provided in addition to the targeted eHealth intervention, as ehealth intervention may not be designed as stand-alone intervention. This includes training sessions and support [1]. It may be necessary to distinguish between the level of training required for the trial, and the level of training for a routine application outside of a RCT setting (discuss under item 21 – generalizability.

	1	2	3	4	5	
subitem not at all important	○	○	○	○	○	essential

Does your paper address subitem 5-xii? *

Copy and paste relevant sections from the manuscript (include quotes in quotation marks "like this" to indicate direct quotes from your manuscript), or elaborate on this item by providing additional information not in the ms, or briefly explain why the item is not applicable/relevant for your study

There were no co-interventions. "Intervention arm participants completed a 45-minute informational onboarding session, including instructions on the interface, technology, and game content".

6a) Completely defined pre-specified primary and secondary outcome measures, including how and when they were assessed

Does your paper address CONSORT subitem 6a? *

Copy and paste relevant sections from the manuscript (include quotes in quotation marks "like this" to indicate direct quotes from your manuscript), or elaborate on this item by providing additional information not in the ms, or briefly explain why the item is not applicable/relevant for your study

This paper presents data on secondary outcomes only. Primary outcomes are discussed in a separate publication.

"All participants completed a self-administered behavioral survey at T1, T2, and T3. The English language survey was completed at the KEMRI offices, using the audio computer-assisted self-interview (ACASI) system."

"The behavioral survey assessed mediators associated with age at onset of sexual activity and condom use at sexual debut, including knowledge, self-efficacy, risk assessment, perceived social norms, attitudes, and behavioral intentions. Thematic areas included puberty, sex, relationships, peer pressure, condom use, HIV, STIs, pregnancy, and alcohol and drugs."

"The game software automatically generates a user log file that records all in-app activity. Each user interaction is time-stamped, allowing for calculation of time spent on specific components of the game, as well as total exposure time."

"Intervention arm participants (n=27) and their parents (n=22) took part in FGDs (n=8) between T2 and T3. The four adolescent focus groups were stratified by age (11-12 and 13-14 years) and gender of the study child; the four parent focus groups were stratified by the age of the study child. Questions in postintervention discussions with participants included what they had learned from the game. Parental focus group questions also included how their children had played the game and communicated about it and with whom."

"Preliminary cleaning of survey data was conducted in MS Excel, with additional cleaning and all analyses completed using SAS version 9.4 (SAS Institute Inc., Cary, NC, USA). All control arm participants were included in analyses. One participant from the intervention arm was removed from analyses of effect at T2 due to delayed completion of the T2 survey. His data were [illegible]

retained for T1-T3 analyses, as he completed all other study activities on time. Descriptive statistics on demographic questions and game feedback questions were computed. Changes in behavioral mediators of sexual behavior from baseline (T1) were compared between the two study arms at T2 and T3 in an intent-to-treat analysis, using two-tailed two-sample t tests on individual survey items, as well as domain-level composite scores. This approach was used to identify both which theoretical mediators and which thematic areas were influenced by the intervention. Composite scores (eg, knowledge) were calculated as the equally weighted sum of the individual items within that domain (or thematic area) for which there were objectively correct or incorrect answers. In composite scores, each correct answer was worth 1 point. Analyses were conducted across the whole sample, as well as stratified by age and gender the participants."
"Data from the phone log files were downloaded as .txt files and converted into Excel files, and exposure time was calculated from time stamps. Focus group transcripts were translated into English and uploaded to MAXQDA 2018 (VERBI Software, Berlin, Germany), where they were labeled with inductive and deductive codes by two coders. The data were analyzed thematically and compared across demographics."

6a-i) Online questionnaires: describe if they were validated for online use and apply CHERRIES items to describe how the questionnaires were designed/deployed

If outcomes were obtained through online questionnaires, describe if they were validated for online use and apply CHERRIES items to describe how the questionnaires were designed/deployed [9].

	1	2	3	4	5	
subitem not at all important	○	○	○	○	○	essential

Does your paper address subitem 6a-i?

Copy and paste relevant sections from manuscript text

Not applicable

6a-ii) Describe whether and how “use” (including intensity of use/dosage) was defined/measured/monitored

Describe whether and how “use” (including intensity of use/dosage) was defined/measured/monitored (logins, logfile analysis, etc.). Use/adoption metrics are important process outcomes that should be reported in any ehealth trial.

	1	2	3	4	5	
subitem not at all important	○	○	○	○	○	essential

Does your paper address subitem 6a-ii?

Copy and paste relevant sections from manuscript text

"The game software automatically generates a user log file that records all in-app activity. Each user interaction is time-stamped, allowing for calculation of time spent on specific components of the game, as well as total exposure time."

"Data from the phone log files were downloaded as .txt files and converted into Excel files, and exposure time was calculated from time stamps."

6a-iii) Describe whether, how, and when qualitative feedback from participants was obtained

Describe whether, how, and when qualitative feedback from participants was obtained (e.g., through emails, feedback forms, interviews, focus groups).

	1	2	3	4	5	
subitem not at all important	○	○	○	○	○	essential

Does your paper address subitem 6a-iii?

Copy and paste relevant sections from manuscript text

"Intervention arm participants (n=27) and their parents (n=22) took part in FGDs (n=8) between T2 and T3. The four adolescent focus groups were stratified by age (11-12 and 13-14 years) and gender of the study child; the four parent focus groups were stratified by the age of the study child. Questions in postintervention discussions with participants included what they had learned from the game. Parental focus group questions also included how their children had played the game and communicated about it and with whom."
"Focus group transcripts were translated into English and uploaded to MAXQDA 2018 (VERBI Software, Berlin, Germany), where they were labeled with inductive and deductive codes by two coders. The data were analyzed thematically and compared across demographics."

6b) Any changes to trial outcomes after the trial commenced, with reasons

Does your paper address CONSORT subitem 6b? *

Copy and paste relevant sections from the manuscript (include quotes in quotation marks "like this" to indicate direct quotes from your manuscript), or elaborate on this item by providing additional information not in the ms, or briefly explain why the item is not applicable/relevant for your study

No

7a) How sample size was determined

NPT: When applicable, details of whether and how the clustering by care provides or centers was addressed

7a-i) Describe whether and how expected attrition was taken into account when calculating the sample size

Describe whether and how expected attrition was taken into account when calculating the sample size.

	1	2	3	4	5	
subitem not at all important	○	○	○	○	○	essential

Does your paper address subitem 7a-i?

Copy and paste relevant sections from manuscript title (include quotes in quotation marks "like this" to indicate direct quotes from your manuscript), or elaborate on this item by providing additional information not in the ms, or briefly explain why the item is not applicable/relevant for your study

This is not included in the manuscript as it is a feasibility trial and it was not powered to detect effects of the intervention, but merely intended to assess directionality of effect.

7b) When applicable, explanation of any interim analyses and stopping guidelines

Does your paper address CONSORT subitem 7b? *

Copy and paste relevant sections from the manuscript (include quotes in quotation marks "like this" to indicate direct quotes from your manuscript), or elaborate on this item by providing additional information not in the ms, or briefly explain why the item is not applicable/relevant for your study

Not applicable

8a) Method used to generate the random allocation sequence

NPT: When applicable, how care providers were allocated to each trial group

Does your paper address CONSORT subitem 8a? *

Copy and paste relevant sections from the manuscript (include quotes in quotation marks "like this" to indicate direct quotes from your manuscript), or elaborate on this item by providing additional information not in the ms, or briefly explain why the item is not applicable/relevant for your study

"Randomization, stratified by the school attended by the participant, gender, and age, was undertaken using a coin flip by a blinded research team member. Within each school, gender, and age block of participants, coin flips were repeated until participants were equally distributed between the two study arms"

8b) Type of randomisation; details of any restriction (such as blocking and block size)

Does your paper address CONSORT subitem 8b? *

Copy and paste relevant sections from the manuscript (include quotes in quotation marks "like this" to indicate direct quotes from your manuscript), or elaborate on this item by providing additional information not in the ms, or briefly explain why the item is not applicable/relevant for your study

"Randomization, stratified by the school attended by the participant, gender, and age, was undertaken using a coin flip by a blinded research team member. Within each school, gender, and age block of participants, coin flips were repeated until participants were equally distributed between the two study arms"

9) Mechanism used to implement the random allocation sequence (such as sequentially numbered containers), describing any steps taken to conceal the sequence until interventions were assigned

Does your paper address CONSORT subitem 9? *

Copy and paste relevant sections from the manuscript (include quotes in quotation marks "like this" to indicate direct quotes from your manuscript), or elaborate on this item by providing additional information not in the ms, or briefly explain why the item is not applicable/relevant for your study

"Assignments were revealed to participants after they had completed the baseline assessment."

10) Who generated the random allocation sequence, who enrolled participants, and who assigned participants to interventions

Does your paper address CONSORT subitem 10? *

Copy and paste relevant sections from the manuscript (include quotes in quotation marks "like this" to indicate direct quotes from your manuscript), or elaborate on this item by providing additional information not in the ms, or briefly explain why the item is not applicable/relevant for your study

"Randomization [...] was undertaken [...] by a blinded research team member" Enrollment procedures were undertaken by other team members. Allocation was revealed by team members not involved in randomization but involved in enrollment.

11a) If done, who was blinded after assignment to interventions (for example, participants, care providers, those assessing outcomes) and how

NPT: Whether or not administering co-interventions were blinded to group assignment

11a-i) Specify who was blinded, and who wasn't

Specify who was blinded, and who wasn't. Usually, in web-based trials it is not possible to blind the participants [1, 3] (this should be clearly acknowledged), but it may be possible to blind outcome assessors, those doing data analysis or those administering co-interventions (if any).

	1	2	3	4	5	
subitem not at all important	○	○	○	○	○	essential

Does your paper address subitem 11a-i? *

Copy and paste relevant sections from the manuscript (include quotes in quotation marks "like this" to indicate direct quotes from your manuscript), or elaborate on this item by providing additional information not in the ms, or briefly explain why the item is not applicable/relevant for your study

This was not a blinded study

11a-ii) Discuss e.g., whether participants knew which intervention was the “intervention of interest” and which one was the “comparator”

Informed consent procedures (4a-ii) can create biases and certain expectations - discuss e.g., whether participants knew which intervention was the “intervention of interest” and which one was the “comparator”.

	1	2	3	4	5	
subitem not at all important	○	○	○	○	○	essential

Does your paper address subitem 11a-ii?

Copy and paste relevant sections from the manuscript (include quotes in quotation marks "like this" to indicate direct quotes from your manuscript), or elaborate on this item by providing additional information not in the ms, or briefly explain why the item is not applicable/relevant for your study

The control group received no intervention so all involved knew which was "the intervention of interest".

11b) If relevant, description of the similarity of interventions

(this item is usually not relevant for ehealth trials as it refers to similarity of a placebo or sham intervention to a active medication/intervention)

Does your paper address CONSORT subitem 11b? *

Copy and paste relevant sections from the manuscript (include quotes in quotation marks "like this" to indicate direct quotes from your manuscript), or elaborate on this item by providing additional information not in the ms, or briefly explain why the item is not applicable/relevant for your study

Not applicable

12a) Statistical methods used to compare groups for primary and secondary outcomes

NPT: When applicable, details of whether and how the clustering by care providers or centers was addressed

Does your paper address CONSORT subitem 12a? *

Copy and paste relevant sections from the manuscript (include quotes in quotation marks "like this" to indicate direct quotes from your manuscript), or elaborate on this item by providing additional information not in the ms, or briefly explain why the item is not applicable/relevant for your study

"Descriptive statistics on demographic
questions and game feedback questions were computed.
Changes in behavioral mediators of sexual behavior from
baseline (T1) were compared between the two study arms at T2
and T3 in an intent-to-treat analysis, using two-tailed two-sample
t tests on individual survey items, as well as domain-level
composite scores."

12a-i) Imputation techniques to deal with attrition / missing values

Imputation techniques to deal with attrition / missing values: Not all participants will use the intervention/comparator as intended and attrition is typically high in ehealth trials. Specify how participants who did not use the application or dropped out from the trial were treated in the statistical analysis (a complete case analysis is strongly discouraged, and simple imputation techniques such as LOCF may also be problematic [4]).

	1	2	3	4	5	
subitem not at all important	○	○	○	○	○	essential

Does your paper address subitem 12a-i? *

Copy and paste relevant sections from the manuscript (include quotes in quotation marks "like this" to indicate direct quotes from your manuscript), or elaborate on this item by providing additional information not in the ms, or briefly explain why the item is not applicable/relevant for your study

There was no attrition.

12b) Methods for additional analyses, such as subgroup analyses and adjusted analyses

Does your paper address CONSORT subitem 12b? *

Copy and paste relevant sections from the manuscript (include quotes in quotation marks "like this" to indicate direct quotes from your manuscript), or elaborate on this item by providing additional information not in the ms, or briefly explain why the item is not applicable/relevant for your study

"Changes in behavioral mediators of sexual behavior from baseline (T1) were compared between the two study arms at T2 and T3 in an intent-to-treat analysis, using two-tailed two-sample t tests on individual survey items, as well as domain-level composite scores.[...] Analyses were conducted across the whole sample, as well as stratified by age and gender the participants."

X26) REB/IRB Approval and Ethical Considerations [recommended as subheading under "Methods"] (not a CONSORT item)

X26-i) Comment on ethics committee approval

	1	2	3	4	5	
subitem not at all important	○	○	○	○	○	essential

Does your paper address subitem X26-i?

Copy and paste relevant sections from the manuscript (include quotes in quotation marks "like this" to indicate direct quotes from your manuscript), or elaborate on this item by providing additional information not in the ms, or briefly explain why the item is not applicable/relevant for your study

"The study was approved by the Emory University and Kenya Medical Research Institute (KEMRI) Institutional Review Boards"

x26-ii) Outline informed consent procedures

Outline informed consent procedures e.g., if consent was obtained offline or online (how? Checkbox, etc.?), and what information was provided (see 4a-ii). See [6] for some items to be included in informed consent documents.

	1	2	3	4	5	
subitem not at all important	○	○	○	○	○	essential

Does your paper address subitem X26-ii?

Copy and paste relevant sections from the manuscript (include quotes in quotation marks "like this" to indicate direct quotes from your manuscript), or elaborate on this item by providing additional information not in the ms, or briefly explain why the item is not applicable/relevant for your study

"Consent and assent were secured at the home of the participants, following an explanation of the study. Parents consented to participate in the postintervention focus groups if their child was randomized to the intervention arm." Additional details will be provided in a separate protocol manuscript: consent was required from one parent at least and the child's assent was also secured for successful enrollment. Staff reviewed the project, responded to questions and rescreened for eligibility prior to consent and assent procedures.

X26-iii) Safety and security procedures

Safety and security procedures, incl. privacy considerations, and any steps taken to reduce the likelihood or detection of harm (e.g., education and training, availability of a hotline)

	1	2	3	4	5	
subitem not at all important	○	○	○	○	○	essential

Does your paper address subitem X26-iii?

Copy and paste relevant sections from the manuscript (include quotes in quotation marks "like this" to indicate direct quotes from your manuscript), or elaborate on this item by providing additional information not in the ms, or briefly explain why the item is not applicable/relevant for your study

In developing the survey: "A draft instrument
was presented to parents for acceptability, then cognitively
tested in 3 rounds with preadolescents to ensure acceptability,
consistent interpretation, and face validity of the questions."
The survey was delivered "using the audio computer-assisted
self-interview (ACASI) system with headphones to protect
privacy."
Gameplay log files were only identified by the participant's study ID, as were survey responses. FGDs were also coded without identifying participant information. Participants and parents were told to contact study staff with any concerns. Study staff were trained to recognize signs of distress during survey taking and to provide counseling and linkage to services if needed.

RESULTS

13a) For each group, the numbers of participants who were randomly assigned, received intended treatment, and were analysed for the primary outcome

NPT: The number of care providers or centers performing the intervention in each group and the number of patients treated by each care provider in each center

Does your paper address CONSORT subitem 13a? *

Copy and paste relevant sections from the manuscript (include quotes in quotation marks "like this" to indicate direct quotes from your manuscript), or elaborate on this item by providing additional information not in the ms, or briefly explain why the item is not applicable/relevant for your study

"We recruited and enrolled 60 adolescent participants. Half of
the participants were allocated to the intervention arm." Also, see Figure 1.

13b) For each group, losses and exclusions after randomisation, together with reasons

Does your paper address CONSORT subitem 13b? (NOTE: Preferably, this is shown in a CONSORT flow diagram) *

Copy and paste relevant sections from the manuscript (include quotes in quotation marks "like this" to indicate direct quotes from your manuscript), or elaborate on this item by providing additional information not in the ms, or briefly explain why the item is not applicable/relevant for your study

There were none. See Figure 1.

13b-i) Attrition diagram

Strongly recommended: An attrition diagram (e.g., proportion of participants still logging in or using the intervention/comparator in each group plotted over time, similar to a survival curve) or other figures or tables demonstrating usage/dose/engagement.

	1	2	3	4	5	
subitem not at all important	○	○	○	○	○	essential

Does your paper address subitem 13b-i?

Copy and paste relevant sections from the manuscript or cite the figure number if applicable (include quotes in quotation marks "like this" to indicate direct quotes from your manuscript), or elaborate on this item by providing additional information not in the ms, or briefly explain why the item is not applicable/relevant for your study

The study phones were collected after the end of the intervention period so this is not applicable here.

14a) Dates defining the periods of recruitment and follow-up

Does your paper address CONSORT subitem 14a? *

Copy and paste relevant sections from the manuscript (include quotes in quotation marks "like this" to indicate direct quotes from your manuscript), or elaborate on this item by providing additional information not in the ms, or briefly explain why the item is not applicable/relevant for your study

This is included in the methods: "We conducted an individually randomized pilot study of the game Tumaini ("hope for the future" in Swahili) in a sample of 60 male and female preadolescents aged 11-14 years in periurban and urban Kisumu, Kenya, between April and June 2017. The intervention was carried out over 16 days during the 3-week school holiday in April 2017 (Figure 1). Assessment was performed via a survey at baseline (T1), immediately postintervention (T2), and at 6 weeks postintervention (T3). Intervention arm participants also took part in focus group discussions (FGDs) after the intervention to provide additional data on the game experience"
Recruitment took place in March 2017.

14a-i) Indicate if critical "secular events" fell into the study period

Indicate if critical "secular events" fell into the study period, e.g., significant changes in Internet resources available or "changes in computer hardware or Internet delivery resources"

	1	2	3	4	5	
subitem not at all important	○	○	○	○	○	essential

Does your paper address subitem 14a-i?

Copy and paste relevant sections from the manuscript (include quotes in quotation marks "like this" to indicate direct quotes from your manuscript), or elaborate on this item by providing additional information not in the ms, or briefly explain why the item is not applicable/relevant for your study

N/A

14b) Why the trial ended or was stopped (early)

Does your paper address CONSORT subitem 14b? *

Copy and paste relevant sections from the manuscript (include quotes in quotation marks "like this" to indicate direct quotes from your manuscript), or elaborate on this item by providing additional information not in the ms, or briefly explain why the item is not applicable/relevant for your study

N/A

15) A table showing baseline demographic and clinical characteristics for each group

NPT: When applicable, a description of care providers (case volume, qualification, expertise, etc.) and centers (volume) in each group

Does your paper address CONSORT subitem 15? *

Copy and paste relevant sections from the manuscript (include quotes in quotation marks "like this" to indicate direct quotes from your manuscript), or elaborate on this item by providing additional information not in the ms, or briefly explain why the item is not applicable/relevant for your study

This is included as Table 3- Participant demographics

15-i) Report demographics associated with digital divide issues

In ehealth trials it is particularly important to report demographics associated with digital divide issues, such as age, education, gender, social-economic status, computer/Internet/ehealth literacy of the participants, if known.

	1	2	3	4	5	
subitem not at all important	○	○	○	○	○	essential

Does your paper address subitem 15-i? *

Copy and paste relevant sections from the manuscript (include quotes in quotation marks "like this" to indicate direct quotes from your manuscript), or elaborate on this item by providing additional information not in the ms, or briefly explain why the item is not applicable/relevant for your study

Participants were asked about smartphone ownership and use. However, access was not an issue for this study since smartphones were provided.

16) For each group, number of participants (denominator) included in each analysis and whether the analysis was by original assigned groups

16-i) Report multiple “denominators” and provide definitions

Report multiple “denominators” and provide definitions: Report N’s (and effect sizes) “across a range of study participation [and use] thresholds” [1], e.g., N exposed, N consented, N used more than x times, N used more than y weeks, N participants “used” the intervention/comparator at specific pre-defined time points of interest (in absolute and relative numbers per group). Always clearly define “use” of the intervention.

	1	2	3	4	5	
subitem not at all important	○	○	○	○	○	essential

Does your paper address subitem 16-i? *

Copy and paste relevant sections from the manuscript (include quotes in quotation marks "like this" to indicate direct quotes from your manuscript), or elaborate on this item by providing additional information not in the ms, or briefly explain why the item is not applicable/relevant for your study

Analyses were based on intent-to-treat assignments. All participants were included in baseline and endline analyses. One participant in the intervention group was excluded from postintervention analyses. This is explained in Tables 4 and 5. All intervention participants initiated gameplay, therefore ITT also represented ToT analyses.

16-ii) Primary analysis should be intent-to-treat

Primary analysis should be intent-to-treat, secondary analyses could include comparing only “users”, with the appropriate caveats that this is no longer a randomized sample (see 18-i).

	1	2	3	4	5	
subitem not at all important	○	○	○	○	○	essential

Does your paper address subitem 16-ii?

Copy and paste relevant sections from the manuscript (include quotes in quotation marks "like this" to indicate direct quotes from your manuscript), or elaborate on this item by providing additional information not in the ms, or briefly explain why the item is not applicable/relevant for your study

Yes. In methods: "Changes in behavioral mediators of sexual behavior from baseline (T1) were compared between the two study arms at T2 and T3 in an intent-to-treat analysis,"

17a) For each primary and secondary outcome, results for each group, and the estimated effect size and its precision (such as 95% confidence interval)

Does your paper address CONSORT subitem 17a? *

Copy and paste relevant sections from the manuscript (include quotes in quotation marks "like this" to indicate direct quotes from your manuscript), or elaborate on this item by providing additional information not in the ms, or briefly explain why the item is not applicable/relevant for your study

Only secondary outcomes are included here as primary outcomes were not related to behavioral outcomes and are presented elsewhere. Results are presented in tables 4 and 5.

"There was no significant difference in the overall baseline scores between the two arms: intervention arm, mean 30.73 (SD 5.32) and control arm, mean 31.13 (SD 4.74); for baseline difference, t58=0.30, P=.76 (see Table 4). The intervention arm saw significantly greater gains in the overall survey scores (mean 8.03, SD 4.46) than the control arm (mean 2.23, SD 3.88) at T3 (t58=−5.38, P<.001). At T3, the intervention arm showed significant gains in knowledge (mean 3.80, SD 2.37) compared with the control arm (mean 0.80, SD 2.14) (t58=−5.14, P<.001). At T3, the intervention arm participants also showed significant sustained increases in self-efficacy scores (mean 2.03, SD 1.83) compared with the control arm (mean 0.63, SD 1.20) (t58=−3.50, P=<.001).

At baseline, participants reported having 7-8 trusted individuals they could turn to for advice. By T3, players had identified a mean of 3.10 additional sources of advice compared with 1.53 for the control arm (t58=−1.19, P=.24).

At T3, the intervention arm participants' score gains for behavioral intentions for risk avoidance and reduction showed significant increases compared with those of the control arm (t58=−2.87, P=.006), although they had not been significant at T2. No significant change was seen in the intervention arm participants' assessment of risk, attitudinal measures, or perceived social norms compared with the control arm.

The intervention arm showed significant increases in survey scores across constructs (eg, knowledge, attitudes, risk assessment, self-efficacy, and behavioral intentions) in the thematic areas of puberty (t58=−3.46, P=.001), HIV (t58=−3.25, P=.002), condoms (t58=−4.06, P=.001), and pressure from adults and peers (t58=−2.41, P=0.02) compared with the control arm (Table 5).

Analyses stratified by gender and age (11-12 year olds vs 13-14 year olds) showed similar patterns in score increases. In

particular, knowledge, self-efficacy, and the thematic domain of condoms showed significant gains in all four subgroups of participants.
Quantitative Game Experience Data
The postintervention survey eliciting participant feedback on the game revealed high subjective measures of the value, relevance, and appeal of the game, as well as participants' perceived gains in self-efficacy to address risk situations. All participants (n=30) indicated that they had learned "a lot" and that the information would be "very useful for the future" (see Table 6). Of these participants, 29 found the information presented to be immediately useful. The overwhelming majority further responded that, after playing, they felt more prepared to handle difficult situations (n=28) and to say no firmly in situations of pressure (n=29). Ratings of the game's appeal were very positive, with most players rating it as "very fun" (n=27) and indicating that they would like to play "much more" (n=28) and would tell their friends to play (n=29)."

17a-i) Presentation of process outcomes such as metrics of use and intensity of use

In addition to primary/secondary (clinical) outcomes, the presentation of process outcomes such as metrics of use and intensity of use (dose, exposure) and their operational definitions is critical. This does not only refer to metrics of attrition (13-b) (often a binary variable), but also to more continuous exposure metrics such as "average session length". These must be accompanied by a technical description how a metric like a "session" is defined (e.g., timeout after idle time) [1] (report under item 6a).

	1	2	3	4	5	
subitem not at all important	○	○	○	○	○	essential

Does your paper address subitem 17a-i?

Copy and paste relevant sections from the manuscript (include quotes in quotation marks "like this" to indicate direct quotes from your manuscript), or elaborate on this item by providing additional information not in the ms, or briefly explain why the item is not applicable/relevant for your study

"Preliminary calculations of exposure indicate that the intervention arm played Tumaini a mean of approximately 27 hours over the 16 days of the intervention."

17b) For binary outcomes, presentation of both absolute and relative effect sizes is recommended

Does your paper address CONSORT subitem 17b? *

Copy and paste relevant sections from the manuscript (include quotes in quotation marks "like this" to indicate direct quotes from your manuscript), or elaborate on this item by providing additional information not in the ms, or briefly explain why the item is not applicable/relevant for your study

N/A

18) Results of any other analyses performed, including subgroup analyses and adjusted analyses, distinguishing pre-specified from exploratory

Does your paper address CONSORT subitem 18? *

Copy and paste relevant sections from the manuscript (include quotes in quotation marks "like this" to indicate direct quotes from your manuscript), or elaborate on this item by providing additional information not in the ms, or briefly explain why the item is not applicable/relevant for your study

The paper indicates that stratified analyses showed similar patterns, however these are not presented due to the small strata sizes

18-i) Subgroup analysis of comparing only users

A subgroup analysis of comparing only users is not uncommon in ehealth trials, but if done, it must be stressed that this is a self-selected sample and no longer an unbiased sample from a randomized trial (see 16-iii).

	1	2	3	4	5	
subitem not at all important	○	○	○	○	○	essential

Does your paper address subitem 18-i?

Copy and paste relevant sections from the manuscript (include quotes in quotation marks "like this" to indicate direct quotes from your manuscript), or elaborate on this item by providing additional information not in the ms, or briefly explain why the item is not applicable/relevant for your study

All intended users initiated gameplay.

19) All important harms or unintended effects in each group

(for specific guidance see CONSORT for harms)

Does your paper address CONSORT subitem 19? *

Copy and paste relevant sections from the manuscript (include quotes in quotation marks "like this" to indicate direct quotes from your manuscript), or elaborate on this item by providing additional information not in the ms, or briefly explain why the item is not applicable/relevant for your study

None were detected or reported.

19-i) Include privacy breaches, technical problems

Include privacy breaches, technical problems. This does not only include physical "harm" to participants, but also incidents such as perceived or real privacy breaches [1], technical problems, and other unexpected/unintended incidents. "Unintended effects" also includes unintended positive effects [2].

	1	2	3	4	5	
subitem not at all important	○	○	○	○	○	essential

Does your paper address subitem 19-i?

Copy and paste relevant sections from the manuscript (include quotes in quotation marks "like this" to indicate direct quotes from your manuscript), or elaborate on this item by providing additional information not in the ms, or briefly explain why the item is not applicable/relevant for your study

N/A

19-ii) Include qualitative feedback from participants or observations from staff/researchers

Include qualitative feedback from participants or observations from staff/researchers, if available, on strengths and shortcomings of the application, especially if they point to unintended/unexpected effects or uses. This includes (if available) reasons for why people did or did not use the application as intended by the developers.

	1	2	3	4	5	
subitem not at all important	○	○	○	○	○	essential

Does your paper address subitem 19-ii?

Copy and paste relevant sections from the manuscript (include quotes in quotation marks "like this" to indicate direct quotes from your manuscript), or elaborate on this item by providing additional information not in the ms, or briefly explain why the item is not applicable/relevant for your study

"Qualitative Game Experience Data
Participants' comments and those of their parents during postintervention FGDs provided context for the gains observed in behavioral survey scores. Participants identified a wide range of knowledge and skills they had gained through playing Tumaini. Puberty, reproductive systems, HIV, STIs, and condom use were mentioned repeatedly. Skills commonly mentioned were saying a strong no, how to use condoms, recognizing and avoiding bad influences, and setting and achieving goals. One female player reported "It taught me how I can abstain from sex and how I can say a firm no to those who are persuading me to have unprotected sex and how I can keep myself away from them" (FGD for females, aged 13-14 years).
Participants reported sharing—or intending to share—what they had learned with their peers. A younger female participant said the game was useful: "If we are under pressure or forced to have sex with someone, I found that very educative and I even teach others" (FGD for females, aged 11-12 years). An older male participant felt confident he could now teach others about condom use: "Tumaini also teaches how to use a condom well and if [my friends] do not know how to use it I would go with them and teach them how to use a condom" (FGD for males, aged 13-14 years).
Many participants described attitudinal learning related to gender, consent, delaying sex, condom use, puberty, and people living with HIV. When asked what he thought of Tumaini, one older male participant responded saying, "the game taught me I do not have to force girls to do something if they do not want to" (FGD for males, aged 13-14 years). Another male participant described Tumaini as "the game that shows girls are as important as the boys are" (FGD for males, aged 13-14 years).
A common theme among both parent and child focus groups was the value of the game in helping children set goals and plan how to achieve them, including when faced with challenges.
Parents reported that their children's newly identified or reinforced goals were encouraging them to study hard and make

good choices in order to be successful. In one child's words, "It helps you plan your future and not make bad choices so that when you grow up you may have a smooth future and a happy family" (FGD for females, aged 11-12 years). This future orientation was presented by parents and children as a key motivator for risk avoidance or risk reduction.
Parents also described how the game had facilitated discussion about HIV and related subjects with their children. Parents reported that participants had sought out adults—parents, older siblings, and teachers—to discuss or validate the information presented in the game. One parent recalled his daughter asking, "Father, so it is true that when out there if a boy calls you to go to where he is you can refuse?" (FGD2 for parents of 13-14 year olds). Another reported, "You know at this stage men may also be interested in this young girl, and if such a thing happens right now I know she would tell me" (FGD1 for parents of 13-14 year olds)."

DISCUSSION

22) Interpretation consistent with results, balancing benefits and harms, and considering other relevant evidence

NPT: In addition, take into account the choice of the comparator, lack of or partial blinding, and unequal expertise of care providers or centers in each group

22-i) Restate study questions and summarize the answers suggested by the data, starting with primary outcomes and process outcomes (use)

Restate study questions and summarize the answers suggested by the data, starting with primary outcomes and process outcomes (use).

	1	2	3	4	5	
subitem not at all important	○	○	○	○	○	essential

Does your paper address subitem 22-i? *

Copy and paste relevant sections from the manuscript (include quotes in quotation marks "like this" to indicate direct quotes from your manuscript), or elaborate on this item by providing additional information not in the ms, or briefly explain why the item is not applicable/relevant for your study

"In this pilot study, we found evidence of significant effects of exposure to a game-based intervention on mediators of sexual risk avoidance and risk reduction, including related knowledge, self-efficacy, and behavioral intentions, in addition to overall survey scores at 6 weeks postintervention."

"FGDs with youth and parents contextualized these quantitative findings within participants' reports of gains in knowledge and skills, increased reflection on and planning for their future, and increased dialogue with parents. The increase in the number of trusted adults identified by participants as sources of information in the surveys was also validated by parents' focus group comments."

"In the behavioral surveys, no significant effect was seen on risk assessment, attitudes, or perceived social norms. However, participants in FGDs mentioned attitudinal learning around themes including gender, consent, delaying sex, and puberty."

"High levels of intrinsic motivation among adolescents and of acceptability to parents are critical for the feasibility of a remotely delivered intervention for this age group. Several sources of evidence triangulate to support Tumaini 's high appeal to participants. An objective indicator of participants' liking of the game is mean exposure, which was over 50% higher than instructed. Enthusiasm for the game in subjective feedback provided immediately postintervention was also reflected in FGDs with participants and with parents."

22-ii) Highlight unanswered new questions, suggest future research

Highlight unanswered new questions, suggest future research.

	1	2	3	4	5	
subitem not at all important	○	○	○	○	○	essential

Does your paper address subitem 22-ii?

Copy and paste relevant sections from the manuscript (include quotes in quotation marks "like this" to indicate direct quotes from your manuscript), or elaborate on this item by providing additional information not in the ms, or briefly explain why the item is not applicable/relevant for your study

This study was a feasibility study and is intended to inform a larger scale RCT to show efficacy of the intervention.

We note, among other things: "A larger study, powered to detect these [changes in attitudes and social norms] effects, is needed in order to better understand whether our narrative-based approach influences attitudes and norms" and "In the context of a larger, longer study, a mediation analysis, drawing on the game log files, will allow us to better identify the active ingredients of this game design."
"A future efficacy study should track behaviors in addition to behavioral mediators and ideally include biomarkers for sexual activity to validate self-report data."

20) Trial limitations, addressing sources of potential bias, imprecision, and, if relevant, multiplicity of analyses

20-i) Typical limitations in ehealth trials

Typical limitations in ehealth trials: Participants in ehealth trials are rarely blinded. Ehealth trials often look at a multiplicity of outcomes, increasing risk for a Type I error. Discuss biases due to non-use of the intervention/usability issues, biases through informed consent procedures, unexpected events.

	1	2	3	4	5	
subitem not at all important	○	○	○	○	○	essential

Does your paper address subitem 20-i? *

Copy and paste relevant sections from the manuscript (include quotes in quotation marks "like this" to indicate direct quotes from your manuscript), or elaborate on this item by providing additional information not in the ms, or briefly explain why the item is not applicable/relevant for your study

"The limitations of this study include the small sample size and limited exposure and follow-up time. A future efficacy study should track behaviors in addition to behavioral mediators and ideally include biomarkers for sexual activity to validate self-report data."

21) Generalisability (external validity, applicability) of the trial findings

NPT: External validity of the trial findings according to the intervention, comparators, patients, and care providers or centers involved in the trial

21-i) Generalizability to other populations

Generalizability to other populations: In particular, discuss generalizability to a general Internet population, outside of a RCT setting, and general patient population, including applicability of the study results for other organizations

	1	2	3	4	5	
subitem not at all important	○	○	○	○	○	essential

Does your paper address subitem 21-i?

Copy and paste relevant sections from the manuscript (include quotes in quotation marks "like this" to indicate direct quotes from your manuscript), or elaborate on this item by providing additional information not in the ms, or briefly explain why the item is not applicable/relevant for your study

This is a feasibility study.

21-ii) Discuss if there were elements in the RCT that would be different in a routine application setting

Discuss if there were elements in the RCT that would be different in a routine application setting (e.g., prompts/reminders, more human involvement, training sessions or other co-interventions) and what impact the omission of these elements could have on use, adoption, or outcomes if the intervention is applied outside of a RCT setting.

	1	2	3	4	5	
subitem not at all important	○	○	○	○	○	essential

Does your paper address subitem 21-ii?

Copy and paste relevant sections from the manuscript (include quotes in quotation marks "like this" to indicate direct quotes from your manuscript), or elaborate on this item by providing additional information not in the ms, or briefly explain why the item is not applicable/relevant for your study

"the duration of the intervention (16 days) was
very brief, which may have limited its potential effects on
mediators of sexual risk. Should the game prove efficacious
and be available for download to parents', older siblings', or
adolescents' own phones, no external time limit would be placed
on gameplay, thereby allowing adolescents to make use of the
intervention at will, potentially maximizing its effects. Once
the game is downloaded, full functionality of the game would
be available without data or internet access."

OTHER INFORMATION

23) Registration number and name of trial registry

Does your paper address CONSORT subitem 23? *

Copy and paste relevant sections from the manuscript (include quotes in quotation marks "like this" to indicate direct quotes from your manuscript), or elaborate on this item by providing additional information not in the ms, or briefly explain why the item is not applicable/relevant for your study

The study "was registered with ClinicalTrials.gov (NCT03054051)."

24) Where the full trial protocol can be accessed, if available

Does your paper address CONSORT subitem 24? *

Cite a Multimedia Appendix, other reference, or copy and paste relevant sections from the manuscript (include quotes in quotation marks "like this" to indicate direct quotes from your manuscript), or elaborate on this item by providing additional information not in the ms, or briefly explain why the item is not applicable/relevant for your study

This will be available as a future publication.

25) Sources of funding and other support (such as supply of drugs), role of funders

Does your paper address CONSORT subitem 25? *

Copy and paste relevant sections from the manuscript (include quotes in quotation marks "like this" to indicate direct quotes from your manuscript), or elaborate on this item by providing additional information not in the ms, or briefly explain why the item is not applicable/relevant for your study

"Acknowledgments
Research reported in this publication was supported by the National Institute of Mental Health of the US National Institutes of Health under Award Number 5R34MH106368 (PI: KW). The content is solely the responsibility of the authors and does not necessarily represent the official views of the National Institutes of Health. This research was also supported by the Emory Center for AIDS Research (P30 AI050409) and the Andrew W. Mellon Foundation. The sponsors paid no role in review and approval of the manuscript for publication."

X27) Conflicts of Interest (not a CONSORT item)

X27-i) State the relation of the study team towards the system being evaluated

In addition to the usual declaration of interests (financial or otherwise), also state the relation of the study team towards the system being evaluated, i.e., state if the authors/evaluators are distinct from or identical with the developers/sponsors of the intervention.

	1	2	3	4	5	
subitem not at all important	○	○	○	○	○	essential

Does your paper address subitem X27-i?

Copy and paste relevant sections from the manuscript (include quotes in quotation marks "like this" to indicate direct quotes from your manuscript), or elaborate on this item by providing additional information not in the ms, or briefly explain why the item is not applicable/relevant for your study

The study sponsor is NIMH. DW led the development of the app for Realtime Associates. The game is owned by Emory University.

About the CONSORT EHEALTH checklist

As a result of using this checklist, did you make changes in your manuscript? *

- ○ yes, major changes
- ○ yes, minor changes
- ◉ no

What were the most important changes you made as a result of using this checklist?

Your answer

How much time did you spend on going through the checklist INCLUDING making changes in your manuscript *

3 hours

As a result of using this checklist, do you think your manuscript has improved? *

- ○ yes
- ◉ no
- ○ Other:

Would you like to become involved in the CONSORT EHEALTH group?

This would involve for example becoming involved in participating in a workshop and writing an "Explanation and Elaboration" document

- ○ yes
- ◉ no
- ○ Other:

Any other comments or questions on CONSORT EHEALTH

Your answer

STOP - Save this form as PDF before you click submit

To generate a record that you filled in this form, we recommend to generate a PDF of this page (on a Mac, simply select "print" and then select "print as PDF") before you submit it.

When you submit your (revised) paper to JMIR, please upload the PDF as supplementary file.

Don't worry if some text in the textboxes is cut off, as we still have the complete information in our database. Thank you!

Final step: Click submit !

Click submit so we have your answers in our database!

SUBMIT

Never submit passwords through Google Forms.

This content is neither created nor endorsed by Google. Report Abuse - Terms of Service - Additional Terms

Google Forms
